# Resistance to Systemic Inflammation and Multi Organ Damage after Global Ischemia/Reperfusion in the Arctic Ground Squirrel

**DOI:** 10.1371/journal.pone.0094225

**Published:** 2014-04-11

**Authors:** Lori K. Bogren, Jasmine M. Olson, JoAnna Carpluk, Jeanette M. Moore, Kelly L. Drew

**Affiliations:** 1 Department of Chemistry and Biochemistry, University of Alaska Fairbanks, Fairbanks, Alaska, United States of America; 2 Institute of Arctic Biology, University of Alaska Fairbanks, Fairbanks, Alaska, United States of America; Duke University Medical Center, United States of America

## Abstract

**Introduction:**

Cardiac arrest (CA) and hemorrhagic shock (HS) are two clinically relevant situations where the body undergoes global ischemia as blood pressure drops below the threshold necessary for adequate organ perfusion. Resistance to ischemia/reperfusion (I/R) injury is a characteristic of hibernating mammals. The present study sought to determine if arctic ground squirrels (AGS) are protected from systemic inflammation and multi organ damage after CA- or HS-induced global I/R and if, for HS, this protection is dependent upon their hibernation season.

**Methods:**

For CA, rats and summer euthermic AGS (AGS-EU) were asphyxiated for 8 min, inducing CA. For HS, rats, AGS-EU, and winter interbout arousal AGS (AGS-IBA) were subject to HS by withdrawing blood to a mean arterial pressure of 35 mmHg and maintaining that pressure for 20 min before reperfusion with Ringers. For both I/R models, body temperature (Tb) was kept at 36.5–37.5°C. After reperfusion, animals were monitored for seven days (CA) or 3 hrs (HS) then tissues and blood were collected for histopathology, clinical chemistries, and cytokine level analysis (HS only). For the HS studies, additional groups of rats and AGS were monitored for three days after HS to access survival and physiological impairment.

**Results:**

Rats had increased serum markers of liver damage one hour after CA while AGS did not. For HS, AGS survived 72 hours after I/R whereas rats did not survive overnight. Additionally, only rats displayed an inflammatory response after HS. AGS maintained a positive base excess, whereas the base excess in rats was negative during and after hemorrhage.

**Conclusions:**

Regardless of season, AGS are resistant to organ damage, systemic inflammation, and multi organ damage after systemic I/R and this resistance is not dependent on their ability to become decrease Tb during insult but may stem from an altered acid/base and metabolic response during I/R.

## Introduction

Multiple organ failure (MOF) after trauma and/or hypoperfusion, such as occurs during systemic ischemia/reperfusion (I/R) injury, has a mortality rate between 30–100%. Currently there is no treatment available that can halt or reverse its progression [1]. Two perpetrators of systemic I/R injury are cardiac arrest (CA) and hemorrhagic shock (HS). During both CA and HS, the body undergoes global ischemia as blood pressure drops below the threshold at which tissues can be adequately perfused. This results not only in a shortage of oxygen, but also a lack of energy substrates and the removal of harmful waste products. If the ischemia persists, ATP levels in a cell can be depleted resulting in the inability to maintain membrane integrity and ion gradients, eventually leading to cell death [2], [3]. Reintroduction of oxygen during reperfusion also causes damage via the formation of reactive oxygen species and subsequent oxidative stress (known as reperfusion injury) [4]. Some studies support a role for lactate and acid base balance as early indicators of oxygen deficit and potential modulators of inflammation [5]–[8]. Other studies suggest that injury to the small intestine initiates an unregulated systemic inflammatory response that propagates MOF [9]–[14]. Comparison of a known I/R intolerant species, such as humans or laboratory rats, to a novel species that resists I/R and the development of a systemic inflammatory response and organ damage/dysfunction after reperfusion may lead to a better understanding of the early events leading to MOF and may identify innovative therapeutics.

Humans and most other laboratory mammals are vulnerable to I/R injury and MOF. However, hibernating mammals have been shown to be resistant to I/R injury in numerous isolated organ preparations compared to laboratory animals. In particular, ground squirrels (GS) have been found to be protected against intestinal I/R injury during the winter season [15], [16]. Livers from winter-season GS were shown to be resistant to cold I/R injury [17] while kidneys from summer- and winter-season GS were protected from cold ischemia/warm reperfusion necrosis and apoptosis ex vivo [17], [18]. In addition, hippocampal slices from interbout arousal (IBA) GS were resistant to I/R [19] and hippocampus and striatum in vivo were protected after global ischemia (CA) in arctic ground squirrels (AGS, *Urocitellusparryii*) [19], [20]. Previous studies have not investigated if GS are protected from the physiological changes leading to MOF after global I/R, and if the animals are resistant to injury, where in the early pathogenesis of MOF do GS differ from I/R injury prone animals?

Here, we test the hypothesis that AGS will be protected from developing a systemic inflammatory responseand organ damage after global I/R injury. We also test the hypothesis that protection levels will vary seasonally (summer active versus winter hibernation) as there are physiological and metabolic alterations that protect the animals from I/R experienced during the periodic arousals from torpor experienced during the hibernation season. Alternatively, since the AGS brain resists I/R injury in both the summer- and winter-season [21], [22], hibernation season may not play an essential role in I/R tolerance in this species of ground squirrel. We first determined if AGS were protected from MOF after I/R in two clinically-relevant models, CA and HS. Next, we utilized the HS model to further delineate probable avenues for protection.

## Materials and Methods

### Animals and ethics statement

AGS were live trapped in Northern Alaska (66°38′N, 149°38′W) as juveniles and were at least one year of age at the time of the experiment. Once at UAF, they were housed at 21–23°C under light conditions based on 69° latitude. CA animals were maintained at those conditions and experiments were conducted in May/June. For HS, in late August, all AGS were moved to a cold room (2°C) and kept on a 4-hour light/20-hour dark cycle to approximate conditions the animals experience year-round in the wild. Food and water were available ad libitum until 20 hours prior to surgery at which time the food was removed but water was available. Female and male AGS (438–1123 g; n = 79) were used in this study based on the availability of wild-caught animals. All summer/euthermic animals were in their post-reproductive/summer active phase as evidenced by body temperature, activity, and lack of spontaneous torpor for at least four weeks. Winter/hibernating animals were distinguished by having regular spontaneous torpor bouts for at least eight weeks prior to the experiments. For IBA animals, arousal was induced 18 hours before the start of the experiment by gentle handling. Prior studies in AGS show that following induced arousal, core body temperature reaches stable euthermic temperature within three hours [23]. All animals were at euthermic core body temperatures at the start of the experiment. Tables S2, S3, S4, S5, S6, S7 delineate characteristics used to assess hibernation season. Male Sprague-Dawley rats (250–320 g for CA and 350–420 g for HS, n = 48) were used as positive, I/R sensitive controls. Rats were purchased from Simonson Laboratory (Gilroy, CA) or from a colony derived from the same source by UAF Animal Resource Center.

Capture and holding of AGS was performed under permit by the Alaska Department of Fish and Game. All animal procedures were performed in accordance with the *Guide for the Care and Use of Laboratory Animals* and approved by the Animal Use and Care Committee of the University of Alaska Fairbanks (UAF) with the exception of two instances. In these cases, pharmaceutical-grade Ringers was not available and Ringers solution was prepared in accordance with the Guide, but without knowledge that use of non-pharmaceutical grade solutions required IACUC approval.

In clinical medicine, it is not standard protocol to administer analgesics after cardiac arrest [24] or HS, in the latter case, due to potential effects on blood pressure. Analgesics were avoided in the present study because analgesics are not used routinely in clinical medicine and because of potential confounding affects on inflammatory processes, CNS injury, and body temperature. Animals would have been euthanized at any point during the procedure or during post-op recovery if complications were encountered that compromised interpretation of experimental results or led to pain or discomfort that was not interpreted as part of the injury model.

### Cardiac arrest

**Figure 1:**
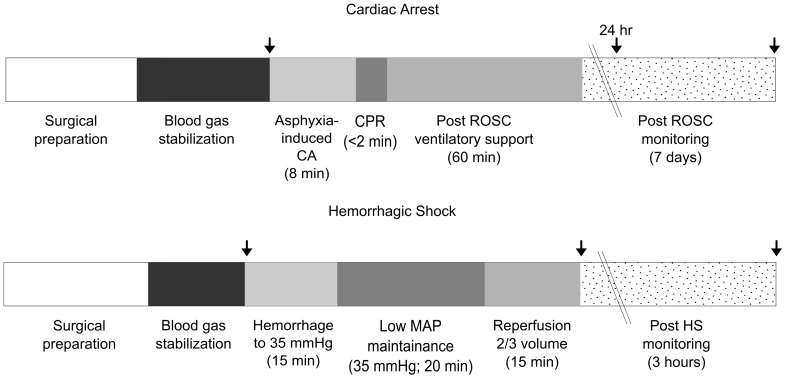
Experimental protocols for ischemia/reperfusion. CA, cardiac arrest; CPR, cardiopulmonary resuscitation; ROSC, return of spontaneous circulation; HS, hemorrhagic shock. Core body temperature and head temperature were maintained between 36.5 and 37.5°C with a warm water blanket under the animal and heat lamps above the animalfrom the start of surgical preparation until the start of post ROSC or post HS monitoring. Arrows indicate blood sampling timepoints.

The asphyxial CA model was employed as described previously [20], [25] (Supporting Materials) except as noted. Positive end-expiratory pressure (PEEP; 1 cm H_2_O) was maintained throughout mechanical ventilation and, because mean arterial pressure (MAP) fell in AGS during anesthesia, baroreceptors along the carotid artery were stimulated by gentle pressure on the neck to increase MAP to normal, unanethetized values of ∼100 mmHg in the AGS prior to asphyxia. MAP was calculated as 2/3 (end systolic pressure)×1/3 (end diastolic pressure). Rats were housed on absorbent pads until ambulatory. Porphyrins were washed from the eyes and pads cleaned daily or more frequently, if indicated. A 50∶50 sucrose∶rodent chow soup mixture was provided. Animals that did not eat spontaneously from the dish were “spoon-fed” with a 3 cc syringe and gavage needle. The gavage needle was placed near the mouth where animals readily licked and swallowed the mixture. Animals were spoon fed in this manner up to 1 mL/kg body weight three times per day. Blood samples for serum and EDTA-plasma were collected from the femoral artery immediately prior to CA (baseline) and 24 hours after return of spontaneous circulation (ROSC), and via cardiac puncture seven days after CA (Fig. 1). Sham animals (SCA) underwent the cardiac arrest surgery but were not disconnected from the ventilator to produce asphyxia. The femoral cannula was externalized at the base of the neck for subsequent blood sampling in unanesthetized animals.

### Hemorrhagic shock

Surgical preparation, cannulation, and monitoring of the animals were the same as for the CA procedure except that both femoral arteries were cannulated and the right femoral artery was not externalized. As with CA, core body temperature and head temperature were maintained at 37±0.5°C from the start of surgical preparation until post HS monitoring. This was achieved with a heated water blanket under the animal and heat lamps placed above the head and back turned on below 36.5°C and off above 37.5°C by thermocouples placed in the temporalis muscle and lower bowel via T-C5C32 temperature controllers (Omega, Stamford, CT).After cannulation and the stabilization of blood gases, the animals were subjected to hemorrhage over a 15 minute period that resulted in a decrease in MAP to ∼35 mmHg. MAP was held at that level for 20 minutes before animals were given intravenous non-lactated Ringers solution equal to 2/3 volume of the blood removed during hemorrhage. During HS, animals were ventilated without adjustment from baseline rate and volume. Reperfusion occurred over a period of 15 minutes at a constant reperfusion rate. After reperfusion, animals were monitored for three hours. Blood samples were collected from the femoral artery immediately prior to HS (baseline), upon end of reperfusion, and from cardiac puncture at the end of the three hour monitoring period (Fig. 1). Sham animals (SHS) were subject to the hemorrhagic shock procedure, but blood was not removed and Ringers was not administered.

### Hemorrhagic shock- 72-hour survival assessment

For 72- hour survival assessment, animals (n = 4 per group; rats, summer/euthermic AGS, and winter/IBA AGS) were returned to home cages (21–23°C) after the three hour monitoring period and observed for physiological deficits(successful formation of a nest in supplied cotton bedding, normal response of moving away from caregiver's hand when placing rodent chow in cage, ability to move about the cage without lethargy or impaired gait, normal feeding, and waste production) at least three times per day beginning 8–12 h after surgery and at least once per day thereafter. To minimize suffering, we employed the shortest time line possible to assess survival and the minimum number of animals required.

### Hemorrhagic shock- isovolumetric

In the isovolumetric studies, animals (n = 4 per group; rats, summer/euthermic AGS, and winter/IBA AGS) had 55% of their calculated total blood volume removed during hemorrhage, regardless of the resultant MAP. Total blood volume was calculated as being equal to 6.5% of the animal's total body weight [26].

Non-lactated Ringers solution (148 mMNaCl, 2 mM CaCl_2_, 4 mMKCl) was obtained commercially (Hospira, Inc.,Lake Forest, IL) for all procedures save two, where it was unavailable. In these instances, Ringers was made with sterile water for injection (Butler & Schein, Columbus, OH) and sterilized by filtering through a sterile 0.2 µm syringe filter (HT Tuffryn Membrane; PALL Life Sciences) prior to use.

Surgeries, I/R events, postoperative monitoring up to three hours (if the animals were not comatose due to the I/R event), and euthanasia (via decapitation)were performed under a surgical plane of isoflurane anesthesia. Analgesics were not administered post-operatively because of their influence or expected influence on inflammatory process and injury following HS and CA [27]–[33].

Experimental design and groups are summarized in Figure 1 and Table S1, respectively. I/R and sham experiments were conducted on alternating days. Naive animals were included as a control for effects of surgery. AGS were matched for sex and hibernation phenotype (Tables S2, S3, S4, S5, S6, S7). Blood chemistries, cytokine levels, quantitative histological analysis, and the morphological analysis of the small intestine were all conducted in a blinded and randomized fashion. Due to technical difficulties, not all animals had samples taken for every parameter measured. The sample size for any given parameter is stated in the table or in the figure legend.

### Blood chemistries and cytokine levels

For glucose and lactate, blood was collected in EDTA and the plasma analyzed with a YSI 2300 STAT glucose/L-lactate analyzer (Yellow Springs, OH). Non-lactated Ringers was used as the reperfusion solution to eliminate external sources of lactate. Analysis of alanine aminotransferase (ALT), aspartate aminotransferase (AST), creatine phosphokinase (CPK), and creatinine was conducted on serum with a Vitros 5600 (Ortho Clinical Diagnostics, Rochester, NY) using standard methods. Base excess (BE), bicarbonate (HCO_3_^−^),pH, P_CO2_, and P_O2_ levels were measured with i-STAT CG8+ cartridges (Abbot, Princeton, NJ) using whole blood. Fold changes in cytokine levels in EDTA-plasma (snap frozen in liquid nitrogen after collection and stored at −80°C) were determined using the Rat Cytokine 10-Plex Panel (no. LRC0002) from Invitrogen (Grand Island, NY) on the Luminex 200 System (Austin, TX)and calculated according to the formula [(pg/mL after HS)−(pg/mL before HS)]/(pg/mL before HS).

### Quantitative histological analysis

Immediately following euthanasia, tissues were collected and suspended in 10% buffered formalin and sent to VDx Pathology (Davis, CA) for trimming and processing into histological slides for evaluation by a veterinary pathologist. At VDx, the tissues were examined and graded according to a standard format based on a dictionary of changes specific for each tissue (Tables S8 and S9; LABCAT, Lawrenceville, NJ, USA). The subjective evaluations of change in each tissue were converted into a system of numerical scoring that provided 16 levels of injury for each type of change evaluated for the extent of lesion size and for degree of tissue change. These data were summarized for each tissue to provide a severity summary score that permitted statistical comparison of changes between species.

### Morphological analysis of small intestine

Small intestine was formalin fixed, blocked in paraffin, sectioned and stained with hematoxlyn and eosin. From each slide, five fields with a total of ∼50 villi from each animal were analyzed using light microscopy at 2–10× in a blinded fashion. The length of each villi was measured using MetaMorph (Molecular Devices, Sunnyvale, CA) and was assessed a damage score as previously described [34]. Briefly, 0 = normal villi, 1 = mild damage with vacuolation at villus tip, 2 = increased space at tip, lifting of epithelial layer from the lamina propia, 3 = moderate damage with massive lifting of subepithluium and vacuolation to the midpoint of the villi, 4 = vacuolation to the base of the villi, 5 = severe damage with mucosal ulceration and loss of villi structure.

### Statistics

All data is expressed as mean±SEM. Statistical analysis was performed using SAS v. 9.1 (Cary, NC). Parameters monitored over time were analyzed via ANOVA with repeated measures. Other parameters were evaluated using a 2-way ANOVA. All significant interaction effects in ANOVAs were followed by Tukey's post-hoc tests. Statistical significance was considered to be a *p* value of <0.05.

## Results

### Cardiac arrest

**Figure 2:**
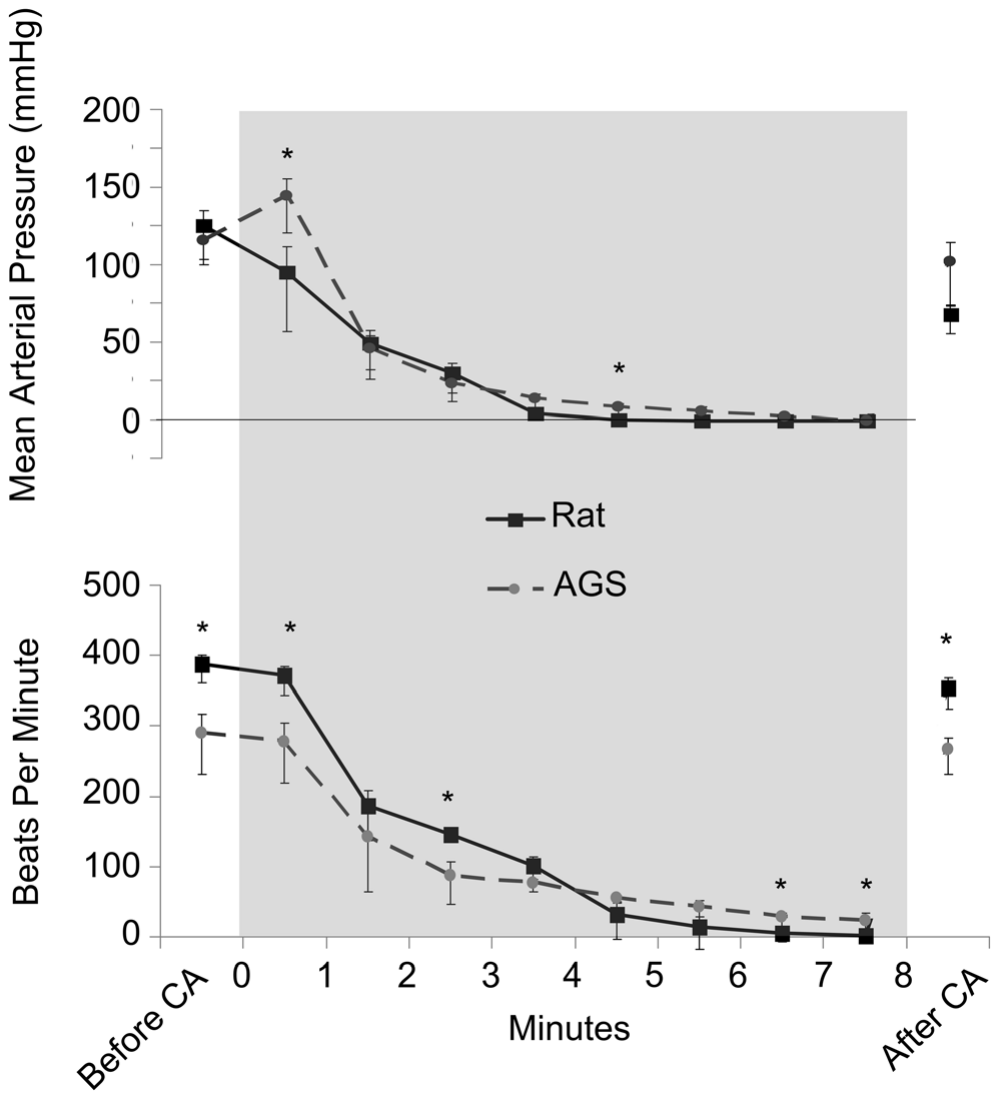
The mean arterial blood pressure and average heart rate decrease during cardiac arrest. Both mean arterial pressure (MAP; top) and heart rate (HR; bottom) decrease similarly for both AGS and rat and return to near normal after CA. Darkened area represents period of asphyxia. MAP and HR were recorded immediately before asphyxiation (Before CA) and one hour after CA (After CA). Data shown as mean ± SEM, * Tukey, *p*<0.05 between rat and AGS. For both groups n = 5.

To test the hypothesis that AGS resist physiological changes leading to MOF after I/R, we first determined if the severity of ischemia during asphyxia-induced CA was similar between rats and AGS. MAP decreased to <20 mmHg [35] for both species after 3.5 minutes of asphyxiation and remained under 20 mmHg for the duration of the arrest (Fig. 2). After ROSC, MAP increased in both species. The AGS had a significant increase in MAP immediately following the onset of asphyxia compared to the rats (*p* = 0.0119, ANOVA, time×species). Despite the increase in MAP at the start of asphyxiation, the AGS had similar MAPs for the remainder of the asphyxiation/CA period. After onset of asphyxia, functional heart beat, as determined by a plusitile increase in systolic and MAP, became uncoupled from cardiac contraction noted on the ECG (pulseless electrical activity). Functional heart beats used to assess heart rate (HR) were therefore indicated by a transient increase in systolic pressure. HR for AGS and rats declined in a similar manner after onset of asphyxia (Fig. 2) however, heart beats persisted in AGS longer than in rat (*p*<0.0001, ANOVA, time×species). By 6.5 minutes into asphyxiation, four out of five rats had a HR of 0, whereas only one AGS had a HR of 0 at 8 minutes. HR in the other AGS remained above 10 bpm throughout the period of asphyxia. Both AGS and rats regained pre-CA HR by one hour after CA.

We next assessed if recovery of basic physiological parameters following ROSC were similar between rats and AGS. Rats remained comatose for several hours after CA while AGS were conscious and responsive. There were no statistically significant differences in whole blood pH, P_CO2_, P_O2_, HCO_3_^−^, or BE before and after ischemia in AGS or rats as shown in Table 1. AGS had a higher BE than rats before and after CA (*p* = 0.0486, ANOVA, main effect of species).

**Table 1:** Physiological parameters returned to pre cardiac arrest values in AGS and rats after cardiac arrest.

		Ischemia	
Group	Variable	Before	After
AGS (n = 4–5)	Body Weight	616±29.62	
	pH	7.47±0.01	7.47±0.05
	P_CO2_ mmHg	39.3±1.74	37.84±3.81
	P_O2_ mmHg	54.2±3.41	44.4±1.17
	HCO_3_^−^mmol/L	28.48±0.65	28.53±1.05
	BE mmol/L*	5.00±0.71	4.75±1.49
	Plasma glucose mg/dL	177.4±8.34	213.4±9.20
Rat (n = 5)	Body Weight	265.2±6.67	
	pH	7.44±0.01	7.47±0.02
	P_CO2_ mmHg	38.2±0.83	37.34±0.74
	P_O2_ mmHg	119.4±4.24	114.4±7.02
	HCO_3_^−^mmol/L	25.68±0.57	27.02±0.88
	BE mmol/L*	1.20±0.80	3.60±1.17
	Plasma glucose mg/dL	189.4±21.10	191.25±9.28

Data shown as mean±SEM * *p*<0.05, Tukey, rat versus AGS.

**Figure 3:**
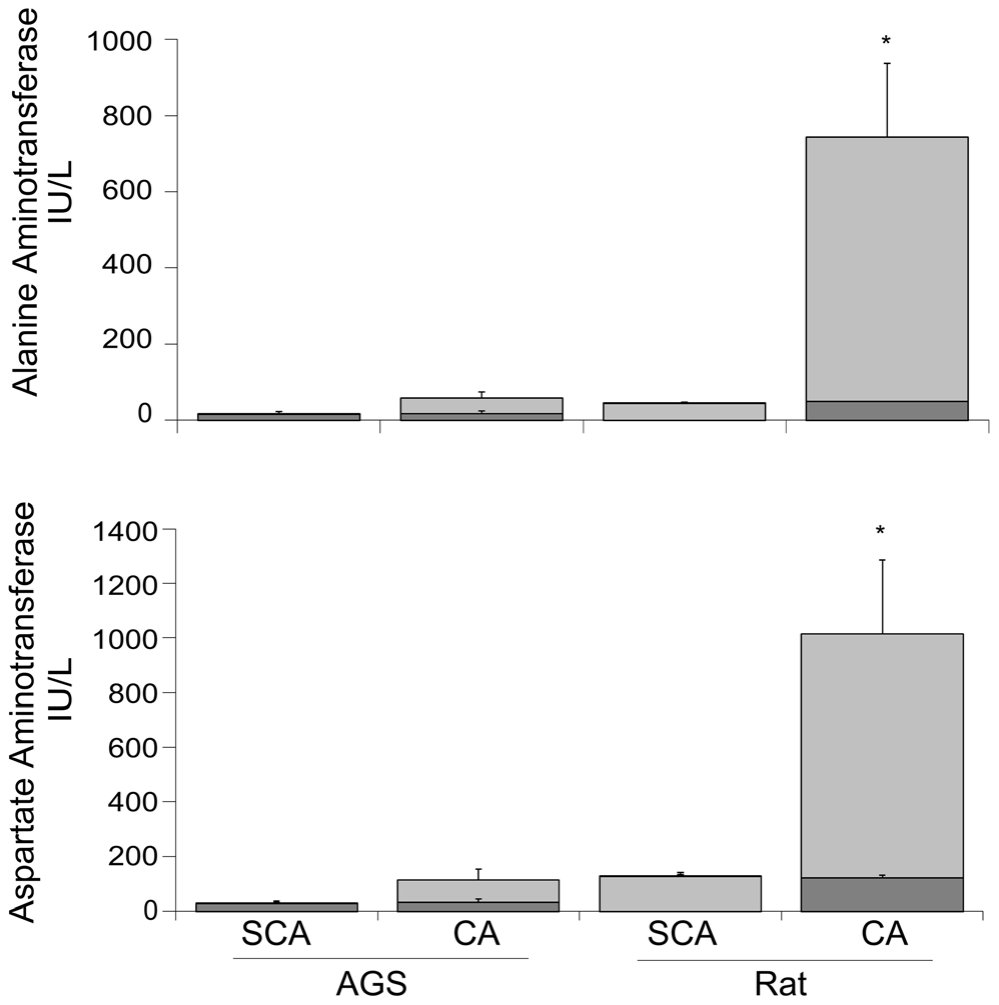
Blood serum markers for liver damage after cardiac arrest. AGS did not show an increase in blood serum markers for liver damage one hour after CA. Rats had an increase in both ALT (top, * Tukey, *p*<0.05) and AST (bottom, * Tukey, *p*<0.05) one hour after CA compared to all other groups. Dark bars indicate baseline values. Light bars are values one hour after CA. Raw data is shown as mean ± SEM. Statistical analysis was done on the difference between baseline and one hour after CA. For all groups n = 5–6.

Lastly for the CA experiments, we sought to determine if organ damage after CA was comparable in the rats versus AGS. Serum markers showed evidence of organ damage after CA in rat but not in AGS (Fig. 3). Plasma levels of both ALT and AST, markers of liver damage, were increased one hour after ROSC in the rats as compared to shams (*p* = 0.0002, ANOVA, group×species×time). AGS did not show liver damage after ROSC as indicated by no significant change in ALT and AST levels. AGS and rats both tended to have an increase in creatine phosphokinase 24 hours after CA which is consistent with heart injury. Lactate dehydrogenase also tended to increase after CA in the rats; indicative of systemic cellular damage. However, due to technical problems, sample size was insufficient for statistical analysis for these two parameters (Fig. S1).

Histopathological analysis of the livers showed multifocal ischemic necrosis in the rat after CA but not in the AGS (Fig. S2). In the rats, accentuation of zonation was found to be present in all five of the CA animals examined. Few changes were found in this area in the AGS while changes of this type were more frequent in the CA rats (mean severity score of 1.40 for rats and 0.8 for AGS). Four of the CA rats had areas of infarction or necrosis in the liver, with no changes in this type found in the ground squirrels.

Quantitative histopathological analysis of the other organs did not reveal significant organ damage and/or failure in either the rats or AGS in CA versus sham animals (Table S10). Heart, lung, spleen, skeletal muscle (right soleus), pancreas, kidney, stomach, small intestine, and large intestine were all examined for signs of damage. Due to the mild degree of organ damage seen after CA in the rat, an alternative model of clinically-relevant I/R, HS, was employed. In addition, instead of allowing the animals to recover for seven days and possibly repair any ischemic damage, tissues were collected three hours after I/R in the HS model. We also assessed response to HS in the AGS during the summer and winter seasons.

### Hemorrhagic shock

For the HS model, the responses of both summer season (euthermic; EU) and winter season (interbout arousal; IBA) AGS to hemorrhage and reperfusion were compared. AGS-EU had not experienced a spontaneous bout of torpor for at least four weeks, whereas IBA animals had displayed two to five months of spontaneous torpor prior to HS (Tables S4, S5, S6, S7). At the outset of the experiment, the IBA animals were still alert, responsive, and at EU core Tb (∼35°C). HR (∼230–270 BPM) prior to HS also confirmed absence of torpor.

**Figure 4:**
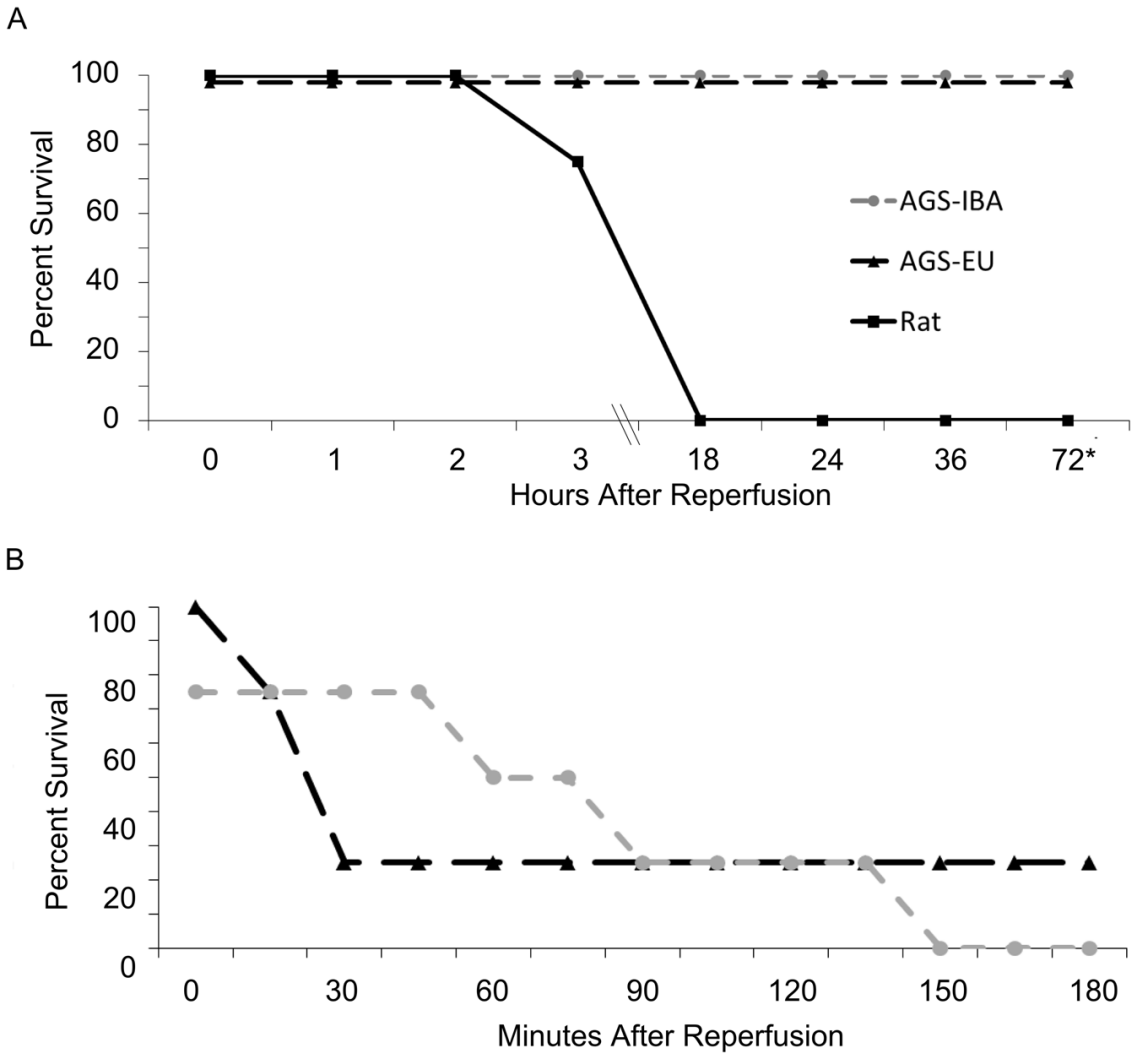
Percent survival after hemorrhagic shock. Extended survival assessed up to 72(MAP ∼35 mmHg for 20 min) in euthermic AGS, interbout arousal AGS, and rats (control; n = 4 for all groups; A). Survival up to three hours after 55–60% blood loss in euthermic- (n = 4) and interbout arousal (n = 4) AGS (B).

To first confirm that HS was a severe model of I/R, survival was assessed in rats and AGS for up to 72 hours after HS. Because parameters for HS vary between laboratories and were optimized in our laboratory for AGS we were unable to compare results in AGS with any other previously published data in rats. All of the animals had similar total blood volume removed as a percent of body weight to achieve a MAP of 35 mmHg (AGS-EU: 31.1±3.5%, AGS-IBA: 29.4±7.3%, Rat: 35.1±3.4%). We found that AGS, regardless of hibernation season, survived for the 72 hour monitoring period after HS with no observable physiological deficit whereas rats did not survive overnight (Fig. 4). Three hours post-resuscitation, the AGS were lethargic, but responsive to stimuli. The rats were unresponsive at this timepoint. Eighteen hours after HS, the AGS had resumed normal appearance and behavior. None of the rats survived to 18 hours post-HS with two of the four still being under anesthesia during the three hour monitoring period when they died (Table S1).

As the AGS were able to survive a ∼30% blood loss, their ability to survive a greater degree of hemorrhage was examined. Both AGS-EU and -IBA were subject to a ∼55% blood loss (AGS-EU: 56.9±1.8%; AGS-IBA: 56.0±0.8%; Fig. 4). The IBA animals survived ∼1 hour after reperfusion, but none survived three hours post resuscitation. The AGS-EU all died within 30 min of resuscitation except for one animal who survived the entire three hour monitoring period. Subsequent studies utilized the isobarameteric model with ∼30% blood loss.

**Figure 5:**
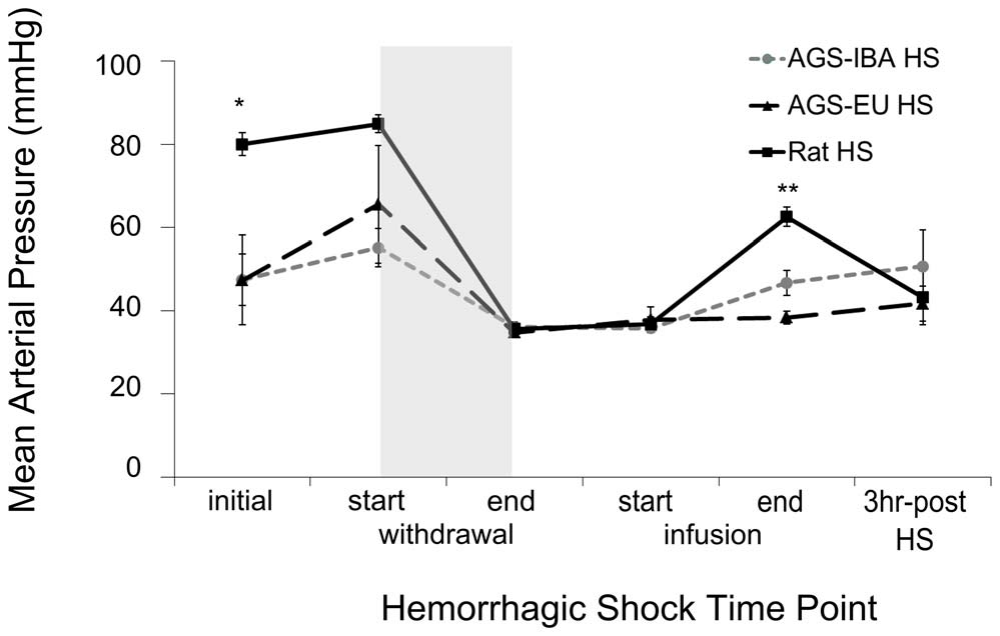
Mean arterial pressure during hemorrhagic shock. Mean arterial pressure decreases to the same mmHg for all groups during HS and is restored to varying degrees with Ringers reperfusion. Shaded region indicates period of hemorrhage. * Tukey, *p*<0.05 between rats and both EU- and IBA-AGS, ** Tukey, *p*<0.05 between all groups. Data shown as mean±SEM, n = 6–7 for all groups.

**Figure 6:**
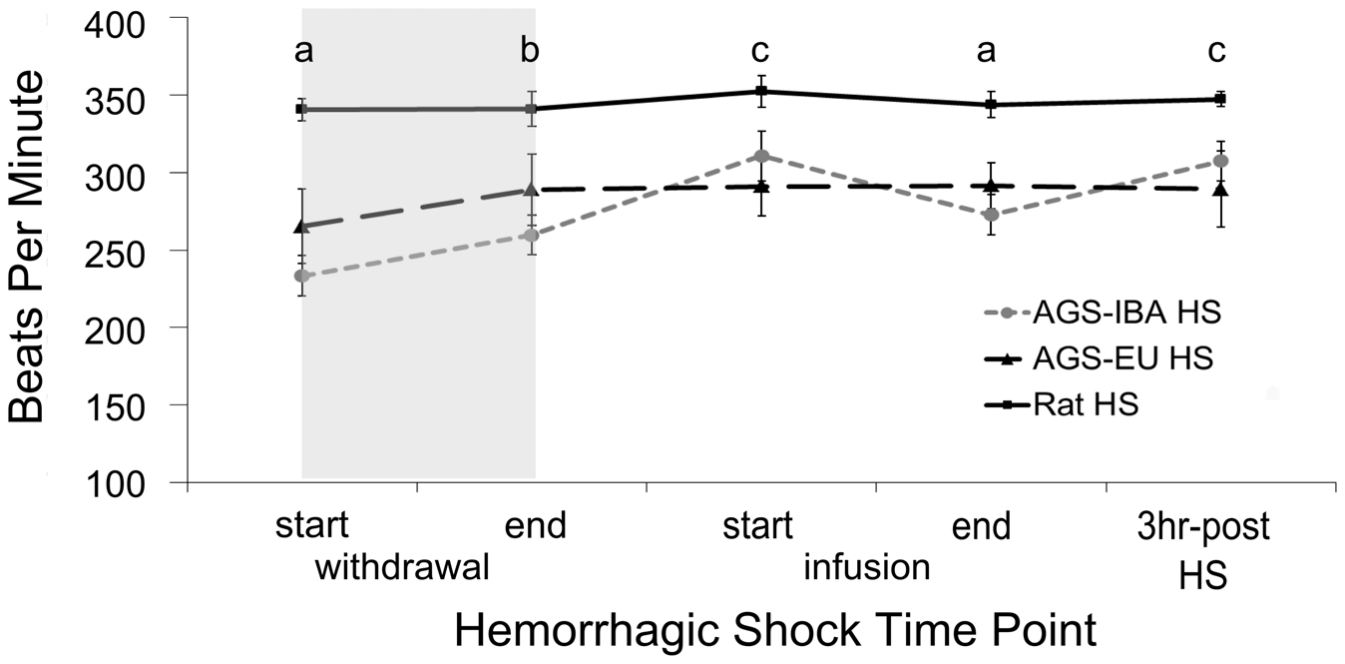
Rats have a higher average heart rate before, during and after hemorrhagic shock than AGS. Shaded region indicates period of hemorrhage. Rats differed from both AGS groups (a, Tukey, p<0.05), just the AGS-IBA (b, Tukey, p<0.05), or AGS-EU (c, Tukey p<0.05). AGS-EU and –IBA did not differ from each other at any timepoint. Data shown as mean±SEM, n = 6–7 for all treatment groups.

We assessed if the animals received similar severity of hemorrhage-induced ischemia. During the hemorrhagic shock experiments, MAP for all animals was brought to 35 mmHg during hemorrhage and held there for 20 minutes before animals were reperfused with non-lactated Ringers solution. Rats had a higher initial MAP than did the AGS (*p* = 0.0027, ANOVA, effect of time×group; Fig. 5). However, percent blood removed, as estimated from body mass, was not affected by initial MAP (AGS-IBA: 33.11±5.39%, AGS-EU: 36.36±4.86%, rat: 34.85±2.01%). Percent change in hematocrit from immediately before hemorrhage to three hours after reperfusion did not differ between groups and were as follows: Rat: −9.9±0.6%; AGS-EU: −9.1±2.7%; AGS-IBA: −8.4±1.8% (mean±SEM, n = 6–7). All three hemorrhaged groups had different MAPs at the end of Ringers reperfusion with rat being the highest (65.0±2.2 mmHg) followed by AGS-IBA (47.1±2.1 mmHg) then AGS-EU as the lowest (38.3±1.5 mmHg; *p*<0.05 Tukey). Rat MAP increased initially but was not sustained. AGS-IBA MAP slowly increased to pre-HS values while AGS-EU did not return to pre-HS levels. MAP in all groups was similarly low three hours post-HS. In contrast to MAP, HR remained constant during hemorrhage and reperfusion for the rats but increased at the end of HS in AGS (*p* = 0.0061, ANOVA, effect of time×group; Fig. 6). Both AGS groups had a lower HR at the start of the experiment and at the end of reperfusion than the rats (*p*<0.05, Tukey). Rats had a higher HR than the AGS-IBA at the start of hemorrhage (*p*<0.05, Tukey) and from the AGS-EU at the end of hemorrhage and three hours post HS (*p*<0.05, Tukey). AGS-IBA and AGS-EU were not statistically different at any timepoint measured during the experiment (*p*≥0.05, Tukey).

Next we asked if hemorrhage and reperfusion had a differential effect on systemic physiology between AGS-IBA, AGS-EU, and/or laboratory rats by monitoring several physiological parameters at the start of hemorrhage (baseline), end of reperfusion, and three hours after I/R (Fig. 1). Table 2 lists the physiological parameters and the corresponding values obtained. Physiological parameters, except for P_CO2_, changed for all groups during hemorrhage and resuscitation. All groups showed a decrease in pH over time (*p*<0.0001, ANOVA, main effect of time). AGS (IBA and EU) had a higher pH than rat (*p* = 0.0009, ANOVA, main effect of group). BE also decreased over time (*p*<0.001, ANOVA, main effect of time) with AGS having a higher BE than rats (*p* = 0.0003, ANOVA, main effect of group). Bicarbonate levels decreased over time for all groups (*p*<0.0001, ANOVA, main effect of time). P_CO2_ and P_O2_ remained unchanged over time with rats having a higher P_O2_ than either group of AGS (*p*<0.001, ANOVA, main effect of group). AGS-EU had lower initial blood glucose than did rats (*p*<0.001, ANOVA, effect of time×group). AGS-IBA had higher initial blood glucose than AGS-EU and higher blood glucose than both AGS-EU and rats at the end of reperfusion and three hours afterwards (*p*<0.001, ANOVA, effect of time×group) despite all groups being fasted ∼20 hours prior to surgery.

**Table 2:** Physiological parameters change for all groups during hemorrhage and resuscitation.

		Hemorrhagic Shock		
Group	Variable	Before	End Reperfusion	3 hr After
AGS-IBA (n = 6–9**)	Body Weight	691.14±98.78		
	pH	7.58±0.03^a^	7.52±0.03^a^	7.51±0.03^a^
	P_CO2_ mmHg	35.87±1.87	37.01±2.16	35.74±3.38
	P_O2_ mmHg	53.67±4.37^a^	50.00±4.79^a^	54.00±7.24^a^
	HCO_3_^−^mmol/L	34.32±2.40^a^	31.46±2.47^a^	31.03±1.96^a^
	BE mmol/L	12.50±2.81^a^	8.80±2.87^a^	8.33±2.28^a^
	Plasma glucose mg/dL	189.14±9.04^a^	229.29±11.12^a^	221.43±13.01^a^
AGS-EU (n = 6–7)	Body Weight	662.40±78.45		
	pH	7.51±0.02^a^	7.47±0.02^a^	7. 50±0.02^a^
	P_CO2_ mmHg	43.00±2.10	41.00±2. 71	36.45±3.42
	P_O2_ mmHg	54.67±2.39^a,b^	50.58±5.41^a^	45.17±5.82^a^
	HCO_3_^−^mmol/L	34.60±1.16^a^	31.60±2.54^a^	27.35±1.48^a^
	BE mmol/L	11.50±1.20^a^	7. 50±2.64^a^	4.67±1.20^a^
	Plasma glucose mg/dL	142.83±10.33^b^	164.67±13.35^b^	164.67±18.29^b^
Rat (n = 6–8)	Body Weight	379.88±11.12		
	pH	7.45±0.01^b^	7.36±0.02^b^	7.32±0.02^b^
	P_CO2_ mmHg	36.72±0.73	34.62±1.46	35.02±1.80
	P_O2_ mmHg	112.13±2.07^b^	122.29±5.49^b^	126.33±12.36^b^
	HCO_3_^−^mmol/L	25.27±0.49^b^	19.62±0.33^b^	18.60±0.46^b^
	BE mmol/L	3.00±0.00^b^	−5.00±1.00^b^	−7.00±0.00^b^
	Plasma glucose mg/dL	218.67±8.18^a^	155.33±8.89^b^	114.67±12.16^b^

AGS had higher pH, BE, P_O2_, and HCO_3_^−^ than rats. Plasma glucose levels were higher for AGS-IBA than rat at the end of reperfusion and three hours after reperfusion and AGS-EU were lower than rat at the start of hemorrhage (different letters indicate *p*<0.05, Tukey between groups at that timepoint).

** n = 5 for AGS-IBA P_O2_.

**Figure 7:**
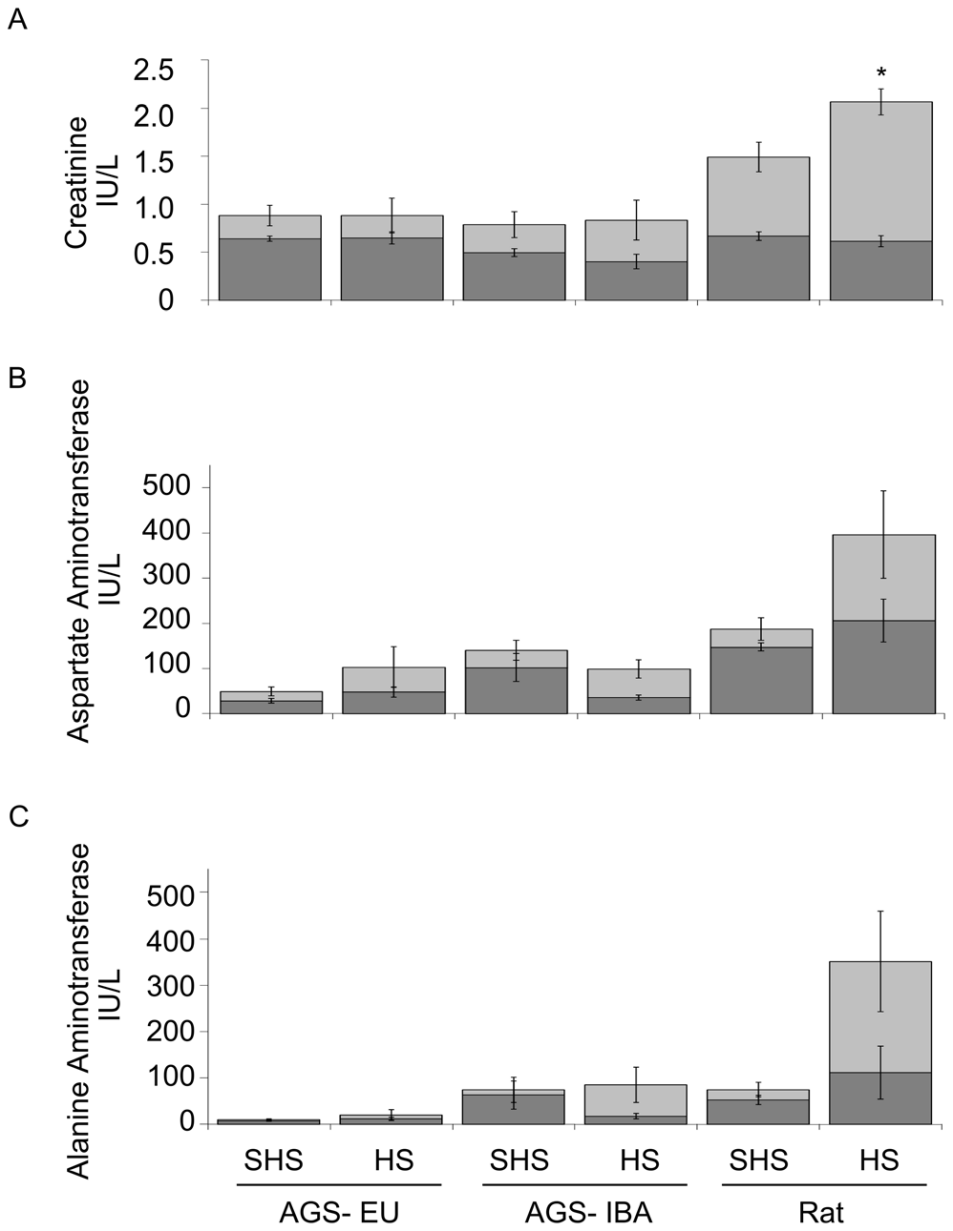
Serum markers of organ damage after hemorrhagic shock. Serum markers indicate that the AGS do not sustain kidney damage as indicated by increased serumcreatinine. Dark areas indicate baseline levels obtained at the start of hemorrhage. Light areas are the levels three hours after end of resuscitation. Raw data is shown and expressed as mean ± SEM Statistical analysis with an ANOVA was performed on the difference between three hours after resuscitation and baseline. *Tukey, *p*<0.05 between SHS and HS; n = 4–8.

Blood markers to assess organ damage showed less kidney damage in AGS than in rat. Rats had a significant increase in serum creatinine, a marker for kidney damage, after HS as compared to baseline (*p*<0.001, ANOVA, main effect of group). Neither AGS-IBA nor –EU had significantly elevated plasma levels of creatinine as compared to baseline after HS (Fig. 7). Rats tended to show elevated levels above baseline of ALT and AST compared to sham animals, indicating liver damage. However, none of the groups had significant increases above baseline in AST or ALT as compared to shams. Quantitative histopathological analysis of the organs showed no evidence of injury at the three hour timepoint. Heart, lung, liver, spleen, and kidney were examined and rated for gross structural and/or cellular damage after HS. Overall, HS did not induce significant histopathological changes in any of these organs for any test group (Table S11). However, relative to naive, changes were noted in the rats' small intestine during the experimental procedure.

As MOF is thought to originate from I/R damage to the small intestine that then instigates a systemic inflammatory response [9]–[14], additional measures were taken to assess intestinal injury from changes in villi length and a damage score. No group of animals showed a HS-induced change in villi length or damage score as compared to SHS. However, in rats, villi length was longer in the naive rats (611.23±28.67 µm) compared to SHS (520.59±36.01 µm) while villi length in SHS and HS were not different, suggesting a mild degree of damage due to surgery (p = 0.0359; ANOVA, main effect of treatment; Fig. S3). Rats also had shorter villi than AGS (*p*>0.0001; ANOVA, main effect of group). However, the overall villi length of any group did not decrease below 500 µM. Quantitative histopathological examination of the intestine did not show any damage due to I/R with the damage score being less than 1.5, even in the hemorrhaged animals. Overall, the rats, both SHS and HS, had a higher damage score than AGS (*p*>0.0001; ANOVA, main effect of group).

**Figure 8:**
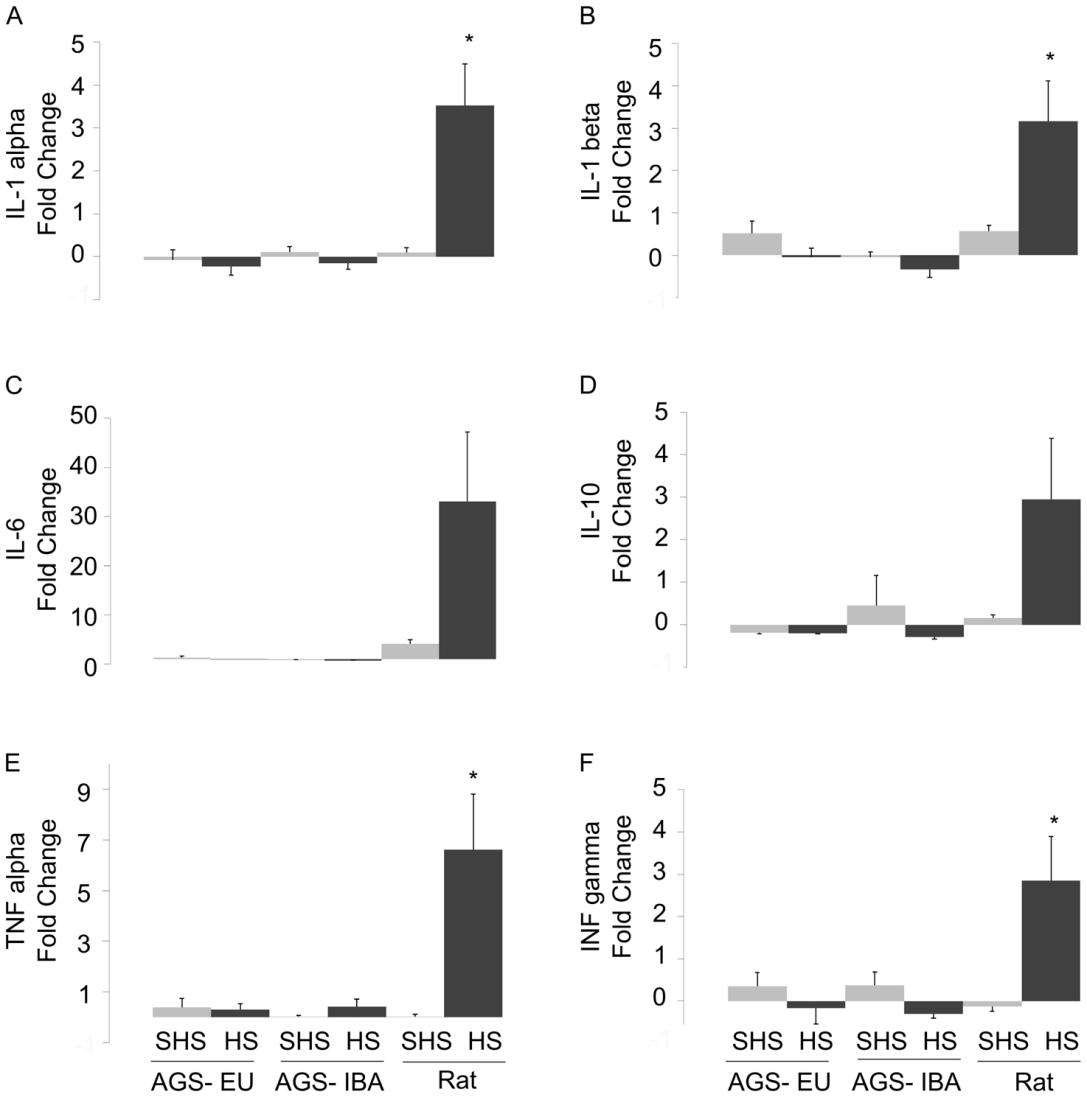
Fold changes in circulating cytokine levels after hemorrhagic shock. AGS do not have a significant fold change in plasma cytokine levels immediately prior to hemorrhage and three hours after resuscitation. Cytokines levels assessed for: IL-1 alpha (A), IL-1 beta (B), IL-6 (C), IL-10 (D), TNF-alpha (E), and INF-gamma (F); *p<0.05, Tukey versus corresponding sham; n = 6–8 for each group.

Without substantial damage to the small intestine, we next determined if changes in plasma levels of inflammatory cytokines showed early signs of an inflammatory response after HS. The results demonstrate that rats displayed an inflammatory response after HS where AGS, in either season, did not. There was a significant difference between HS versus SHS in rats for IL-1 alpha (*p* = 0.0006), IL- beta (*p* = 0.004), TNF-alpha (*p* = 0.0049), and INF gamma (*p* = 0.0029, ANOVA, effects of group×treatment). A similar trend was seen for IL-6 and IL-10 although groups were not statistically different. By contrast, AGS did not demonstrate a systemic inflammatory response after HS-induced I/R. Circulating levels of IL-1 alpha, IL-1 beta, IL-6, IL 10, TNF-alpha, and INF-gamma all remained unchanged after hemorrhage and reperfusion versus sham (Fig. 8). For some cytokines, the levels decreased after hemorrhage. This is most likely due to dilution by Ringers during reperfusion. Total volume of Ringers returned after HS was 5.56 mL, 9.35 mL, and 8.71 mL for rat, AGS-EU, and AGS-IBA, respectively. Levels of circulating cytokines (pg/mL) in naïve animals prior to and three hours after HS are given in Table S12.

**Figure 9:**
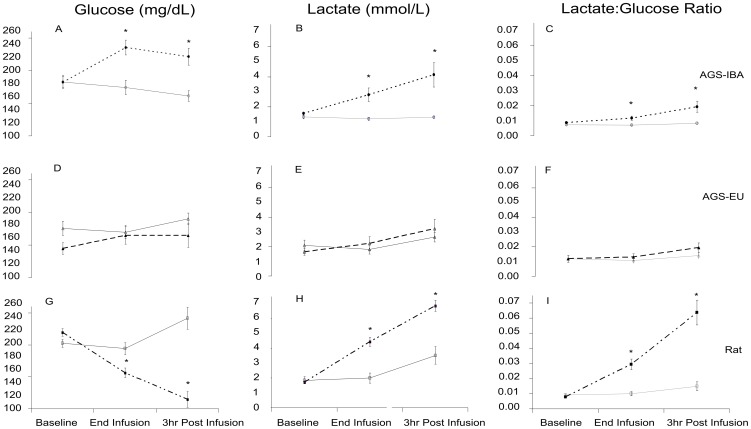
AGS maintain lower blood lactate∶glucose ratio throughout hemorrhagic shock and reperfusion. Blood glucose, lactate, and lactate∶glucose ratio, before, during and after HS Solid lines are SHS, dashed lines are HS. * Tukey, *p*<0.05 between SHS and HS. Naive values for glucose were 162.29±10.94, 153.88±10.69, 205.67±7.78 mg/dL; lactate 1.66±0.29, 1.48±0.25, 1.92±0.20 mmol/L; for AGS-EU, AGS, IBA, and rats, respectively.

Next, we looked at possible cellular mechanisms that may have mitigated the early inflammatory response and mild kidney damage. Hibernators are well known for their ability to suppress their metabolism as well as their ability to switch from a glucose- to fat-based metabolism. The possibility that metabolic alterations present in the AGS may have been associated with their protection against HS-induced organ damage was investigated by monitoring glucose and lactate levels during the HS experimental protocol and afterwards. AGS-IBA had increased levels of plasma glucose while rats' plasma glucose levels decreased during and after HS as compared to sham (*p*<0.0001, ANOVA, effect of group×treatment×time; Fig. 9). AGS-EU had no difference between HS and SHS glucose levels at any timepoint. Concurrently, lactate levels increased during and after I/R as compared to sham in both AGS-IBA and the rat while not differing from sham animals for the AGS-EU group (*p* = 0.005, ANOVA, effect of group×treatment×time). There was no difference in lactate levels between the groups at the start of hemorrhage (*p*>0.05, ANOVA). During hemorrhage, lactate levels rose to a greater extent in the rat (6.88±0.38 mmol/L) than in the AGS-IBA (4.48±0.85 mmol/L). During and after hemorrhage, AGS-IBA and rats had significantly elevated lactate∶glucose ratio compared to sham animals while the AGS-EU HS had ratios comparable to sham animals (*p*<0.0001, ANOVA, effect of group×treatment×time). These results suggest a difference in metabolic response to I/R insult in all three groups with the overall increase in lactate∶glucose being greatest in the rat.

**Figure 10:**
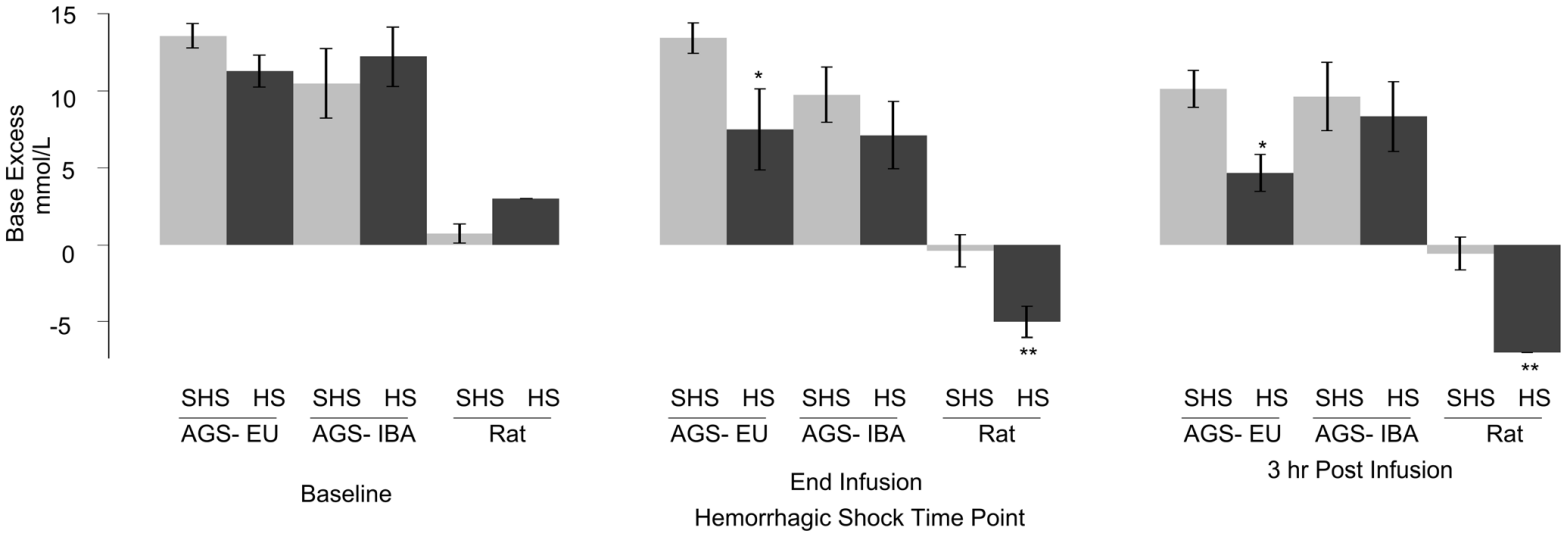
AGS maintain positive whole blood base excess values before, during, and after hemorrhagic shock. All animals initially had positive BE values with the AGS (both seasons) having a starting BE higher than the rats. Data presented as mean±SEM; * Tukey, *p*<0.05 between AGS-EU Sham HS (SHS) and HS, ** Tukey, *p*<0.05 between rat SHS and HS.

To examine the role of acid-base balance in HS-protection, we examined BE levels over the course of hemorrhage and reperfusion. BE values, which correspond to the metabolic component of systemic acid base balance, corroborate a metabolic component to the protection from I/R organ damage observed in the AGS. All animals initially had positive BE values (Fig. 10). The AGS (both seasons) had a starting BE higher than the rats (*p*<0.0001, ANOVA, effect of treatment×time). AGS-IBA had similar BE values compared to sham during and after I/R. In contrast, AGS-EU HS and rat HS had a continued decline compared to sham. From the start of hemorrhage to three hours post resuscitation, all groups had statistically the same overall decrease in BE. However, both AGS-IBA and EU were able to maintain a positive BE for the duration of the experiment, whereas in rats, the decline resulted in a negative BE during and after hemorrhage.

## Discussion

Here we have shown for the first time that resistance to I/R injury in the arctic ground squirrel includes resistance to systemic inflammation and organ damage characteristic of MOF and that this resistance is independent of hibernation season and decrease in Tb. We found that the AGS were protected from physiological changes leading to MOF after both CA and HS. After HS, AGS maintained a positive BE, even up to three hours after reperfusion, and did not have increased levels of circulating cytokines. Overall, the results indicate that AGS protection from I/R injury and MOF stems from metabolic protection based on an altered lactate and BE response to I/R and an absence of systemic inflammation.

Laboratory rats responded to I/R as expected of an I/R vulnerable species in a manner similar to that described for humans with altered acid base balance, systemic inflammation, and markers of organ damage/dysfunction, while AGS resisted I/R injury and organ damage after both CA and HS [12], [36]. Classically, the first indication of the progression of I/R to MOF is an increase in circulating lactate levels and a decrease in BE. Simultaneously, I/R damage to the mucosal layer of the small intestine induces a systemic inflammatory response that then stimulates damage to other organs, leading to their dysfunction and ultimately death [5]–[8].

After HS-induced I/R, the laboratory rats had a pronounced increase in lactate that was even more pronounced when expressed as a fraction of glucose levels. This increase in lactate was accompanied by a decrease in BE to negative values by the end of reperfusion that continued to decline three hours after reperfusion. Although no damage was seen in the rats' small intestine, an inflammatory response, as evidenced by the increase in plasma levels of cytokines, was generated. After I/R, the rats had increased serum markers of kidney damage and were unable to survive eighteen hours post I/R. Conversely, AGS-EU maintained lactate levels and both AGS-IBA and -EU preserved positive BE throughout the experiment. In addition, AGS did not mount an inflammatory response and did not sustain kidneydamage. These findings are important because they demonstrate that the AGS are resistant to organ damage after I/R in a EU state and that the resistance was not due to more downstream effectors of MOF such as damage to the small intestine and systemic inflammation, but most likely in a metabolic or acid/base balance adaptation that is present and evocable throughout the year. Interestingly, in AGS-IBA, lactate and BE after HS showed signs of mild metabolic acidosis and fell mid-way between the response of AGS-EU and rats. This acidosis in AGS-IBA may be due to a combined, “two-hit” effect of HS following the metabolic challenge of arousal [37]. A two-hit phenomenon, as described in clinical literature whereby a severe insult in the form of infection or trauma is hypothesized to prime the host immune system so that a subsequent, relatively trivial insult produces a markedly exaggerated host immune response [38]. Despite this double challenge, AGS-IBA had sufficient buffer to maintain a positive BE and this buffer may have prevented the systemic inflammatory response. This metabolic acid/base buffer may be fundamental to resistance to I/R seen in AGS following CA and HS.

### AGS had no organ damage after CA

AGS had similar responses and duration of CA I/R insult as did the rat controls but were found to have no organ damage after CA. To first determine if AGS were resistant to MOF after I/R, only summer AGS-EU were used, thereby eliminating the seasonality variable and providing the most theoretically I/R intolerant system for the hibernating mammal. Rats had a higher initial resting HR than did the AGS. Although the heart rate of the AGS did not cease, as did the rats, and was significantly higher than the rats for the last two minutes of CA, both groups reached the same MAP of ∼0 mmHg for the last three minutes of asphyxiation. The AGS did have an increase in MAP during the first minutes of asphyxia, but by the second minute of asphyxia, AGS MAP was the same as rats. This increase was not seen in previous studies [20] and could be due to basal MAP manipulations in AGS prior to initiation of asphyxia. This may have had an effect that extended briefly into the asphyxia period. However, aside from the increase in MAP during the first minutes of asphyxia, measurements corresponded to those previously reported in this model [20]. Overall, the MAP of the AGS and rats fell rapidly to <20 mmHg after initiation of the insult.

Rats had an increase in AST and ALT, serum markers for liver damage, 24 hours after CA. Although AGS had a similar duration and extent of low MAP as the rats, they did not show increased serum markers of liver damage. Both of these are enzymes located in liver parenchyma cells and serum levels are raised when liver cells are damaged [39]. AST is also present in red blood cells, as well as in skeletal and cardiac muscle and is therefore not as specific for liver damage as ALT. AST and ALT were both increased to a similar extent in the rats after CA indicating that liver cells had incurred damage. Results from the HS I/R experiments corroborated that the lack of liver damage in the AGS after CA was most likely due to an intrinsic lack of vulnerability to I/R damage present in the AGS rather than a difference in the severity of I/R. In the HS experiments, AGS and rats experienced the same extent and duration of I/R (Fig. 5), but AGS also did not show a trend for increased serum markers for liver damage while the rats did.

In addition to an increase in serum markers for liver damage, rats subjected to CA I/R also displayed multifocal ischemic necrosis in the liver upon histopathological examination. AGS showed no significant organ damage, in liver or other organs, upon histological examination seven days after CA. Dave et al. (2006) demonstrated that AGS were resistant to neuronal damage after CA. In that study, the MAP of the AGS decreased to 0 mmHg by the eight minutes of asphyxia and was not statistically different from rats for the last five minutes of asphyxia. In the present study, AGS MAP also reached 0 mmHg by eight minutes of asphyxia, but declined more slowly than MAP in rat. It may be that in this study, the degree of I/R injury was not as great as previous studies and this could have contributed to the lack of histological organ damage seen in AGS. Additionally, the lack of organ damage could be due to repair mechanisms employed during the seven days between CA and tissue harvest. The duration of ischemia and reperfusion along with the period between I/R and tissue collection vary widely from study to study. Martin et al. [15] and Kurtz et al. [16] had 20 min of ischemia, 60 min of reperfusion followed immediately by tissue collection, Lindell et al. [17] reperfused livers for 60–240 min after isolation (ischemia) as a transplant study. The timepoint for tissue collection of the current CA experiments was set at seven days initially as that is when ischemic damage to brain tissue to best observed and related to previous I/R studies in the lab [20], [25]. However, in the HS model, the tissues were harvested three hours after I/R and no damage was observed at that timepoint. It could be that the HS timepoint was too early to detect damage due to apoptosis. Nevertheless, as the animals survived with no apparent physiological damage for three days after HS-induced I/R, it is unlikely that gross damage to any organ occurred.

Previous studies of CA in the AGS were focused on neuronal damage after global cerebral ischemia and found that the AGS were resistant to such insult [20], [25]. The current study extends those results to include the other major organs being protected from CA I/R.

### Metabolic acid-base component to I/R protection

Hemorrhagic shock was used as a second clinically relevant form of I/R injury to assess the possible mechanisms of resistance against I/R-induced physiological changes leading to organ damage observed in the AGS after CA. In the HS experiments, we first looked at markers of global cellular damage. If cells resist I/R damage, there would be no inflammatory response and no organ damage. One possible means of cellular protection is to reduce acidosis caused by lactate generation under anaerobic metabolism. Previous studies have shown that increased lactate∶glucose ratios are found after pathophysiological/ischemic events and are indicative of poor outcome [40]–[42]. Here we found that rats had an increase in lactate levels during and after HS, as did AGS-IBA. AGS-EU did not have an increase in lactate levels during or after HS. Additionally, the rats had marked decrease in glucose levels over the same time period while the AGS-EU maintained glucose levels and AGS-IBA increased glucose levels. Overall, this indicates that the rats utilized their glucose stores and processed the energy source under anaerobic conditions to produce lactate. The AGS-IBA appear to have been mobilizing or synthesizing glucose and metabolizing it, at least in part, under anaerobic conditions. Increase in lactate in AGS-IBA was unexpected and may reflect increased glucose availability and production of lactate during arousal [37] that when followed by hemorrhage leads to an increase in lactate not seen in AGS-EU. The double hit of arousal followed by hemorrhage may have contributed to lactate accumulation, but was not sufficient to induce a cytokine response in AGS.

The AGS-EU showed no increase in lactate as well as an absence of inflammatory response. Unlike rat and AGS-IBA, AGS-EU did not increase circulating glucose levels. This may indicate that the HS produced a decrease in metabolic rate without a decrease in Tb or a decrease in glucose turnover via aerobic or anaerobic glycolysis. Low glucose turnover could result from a switch in energy substrate from glucose to substrates that cannot be metabolized into lactate, such as D-β-hydroxybutyrate and free fatty acids. In addition, AGS may benefit from greater tissue perfusion than I/R vulnerable species. Although more experiments are needed, the results from the current experiments suggest that, at least in part, AGS-EU may also be able to readily utilize energy sources other than glucose such as free fatty acids and/or D-β-hydroxybutyrate during I/R, reduce their metabolism, and/or benefit from greater tissue perfusion [43], [44].

BE was also monitored during the experiments. BE accounts for the metabolic component of acidosis. When BE is negative, it indicates that the organism is acidic and that a portion of this acidosis has a metabolic basis (e.g. lactic acid build up). Here, BE in hemorrhaged rats was negative by the end of reperfusion and continued to drop until three hours after reperfusion. In contrast, both AGS-IBA and -EU maintained a large positive BE over the course of the experiment. Both the AGS and rats had a decrease in BE over the course of the experiment; however, the AGS started with such a large positive BE that a positive BE was maintained throughout I/R and afterwards. Taken in conjunction with the lactate and glucose data, this implies that AGS may possess a better buffering capacity that enabled them to remain in acid-base balance despite I/R insult. Endogenous buffer against I/R acidosis could not be evaluated in the CA model since exogenous bicarbonate was administered at ROSC. Normalizing BE in rats with exogenous bicarbonate was not sufficient to match outcome in AGS suggesting that metabolic acidosis alone cannot explain the species difference in response.

### AGS small intestine remained undamaged with no subsequent systemic inflammation after HS

MOF after HS has been shown to stem from a systemic inflammatory response to I/R damage to the small intestine that induces the release of non-microbial, inflammation-inducing “danger” molecules (e.g. danger associated molecular pattern molecules; DAMPS) into circulation [13], [14].Here, there was no histopathological damage to the small intestine after HS in the rats or AGS as compared to SHS. There was a greater degree of damage in both the SHS and HS rats compared to AGS. This could be due to a species difference in tissue-specific response to the surgical protocol. Shorter villi length in SHS and HS rat suggests an effect of surgery that may have masked an effect of HS. Nonetheless, the degree of damage seen in the rat intestine (both SHS and HS) was minimal and consisted of mild damage with vacuolation at villus tip. Although there was minimal damage seen in the small intestine of the laboratory rats after HS, there was a significant increase in the level of circulating cytokines, indicating a systemic inflammatory response. The lack of injury in the small intestine may have been due to the timepoint at which it was harvested and the means used to assess damage. As the tissue was collected three hours after reperfusion, there may not have been sufficient time for necrotic and/or apoptotic process to be visible upon histopathological examination. This is unlikely as small intestine is one of the organs most sensitive to I/R where even a delay in preservation after euthanasia can produce visible tissue damage (VDx Pathology, personal communication). The preservation of the small intestine in AGS-EU is contrary to previous studies where I/R produced extensive cellular necrosis and damage to the villi in the mucosal layer [16]. Intestinal response to the surgical preparation seen in both HS and SHS may have masked the I/R-induced injury at the timepoint sampled.

### No organ damage after HS in the AGS

Like small intestine, neither kidney nor liver showed histopathology at the timepoint tested. However, damage was indicated by serum markers for liver damage (AST and ALT) that tended to increase and markers for kidney damage (creatinine) that increased after HS in the rats. In contrast, AGS showed no indication of organ damage from either blood serum markers or histopathological examination, regardless of season.

### Both AGS-IBA and -EU had similar severity of HS and survival rates

To further distinguish the ability of AGS to resist I/R injury, survival after HS was assessed, up to 72 hours. Rats were unable to survive three hours past HS. Conversely, after HS both AGS-IBA and –EU survived with no apparent physiological deficit out to 72 hours. Although both AGS-IBA and –EU had lower initial MAP, the approximate percentage of total blood removed to achieve and maintain a MAP of 35 mmHg was similar to that of the rats (∼35% total blood volume). Previous work in our lab has determined that the unanaesthetized resting MAP of AGS is ∼100 mmHg (unpublished). These data suggest that while the AGS may normally have a MAP close to that of the control rats, they are able to tolerate extended durations of low pressure without adverse effects. This may be an adaptive mechanism that the animals normally utilize during torpor bouts when HR and MAP both plummet to near zero. However, that the animals are able to tolerate this MAP when metabolic rate is not depressed is a novel finding. Previous studies using AGS did not show a reduced MAP under anesthesia. Further studies are needed to determine the cause of the low blood pressure during anesthesia and what role it may play in resistance to I/R injury.

### AGS outcome after global I/R is independent of season or hypothermia

Overall, we found that AGS were resistant to organ damage after I/R in a seasonally-independent manner. In several species, I/R tolerance in isolated organ preparations has only been observed during the winter season. In the previous studies during the winter/hibernation season, ambient temperature was 4–5°C while during the summer/EU season animals were housed at 22°C [15]–[17], [19]. In the present study, designed to assess the effects of a seasonal influence on I/R injury, AGS were housed at 2°C year round to avoid variation in Ta. A Ta of 2°C also approximates burrow temperatures for the species. In the wild, summer AGS spend less than twelve hours above ground each day [45]. Most of their time is spent in subterrestrial burrows where the surrounding soil temperature is between −2°C in May and 5°C in August [46], [47]. Thus, being house at 2°C during the EU/summer season is a more natural environment. Being housed at 20°C may be stressful to the animals; this stress could contribute to less I/R tolerance in the summer season. Nonetheless, 22°C ambient temperature did not appear to affect tolerance to CA in AGS-EU in the present study or in previous studies [25]. Lack of effect of season may also stem from a species difference. AGS are extreme hibernators whose core body temperature can reach −2.9°C during bouts of torpor [48]. The plasticity required for such extreme tolerances of body temperature may also extend to an increased ability to withstand I/R as the animal arouses from sub-freezing temperatures to normothermia. Also, considering the extremely short summers in the Arctic, it may be more time and energy efficient to maintain the biochemical pathways needed in the winter to withstand such perturbations rather than to expend resources to down-regulate and up-regulate pathways solely to be utilized in the summer.

The variable of hypothermia-induced protection was avoided in the present studies as all animals' core and temporalis muscle temperatures were maintained between 36.5 and 37.5°C for the duration of the experiments. This finding is consistent with recent studies of CA-induced I/R in the AGS where AGS-EU were found to be resistant to brain injury after CA even when maintained between 36.5 and 37.5°C during I/R [20], [49].

The present study does not rule out the possibility that some of the differences in response to I/R between AGS and rats are due to the wild versus laboratory-bred nature of the AGS and rat. Additionally, difference in body mass between rats and AGS may have accounted for some of the physiological and survival parameters measured in this study. This possibility is not supported when influence of body mass is considered within AGS, where body mass varied widely. Some AGS had body weights similar to rats yet there was no relationship between body weight and AGS response to I/R. Also, based on this study alone, it cannot be concluded that AGS are resistant to I/R injury in general compared to other mammals, but rather that they are more resistant compared with the laboratory rat. Nonetheless, one goal of the study was to compare a known I/R tolerant species (AGS) to a known I/R intolerant species (rat) and to delineate where the two groups (intolerant versus tolerant) differed in the propagation of injury. Rats, like humans, do not tolerate I/R. Although the laboratory breeding and lifestyle of a rat may influence response to I/R, the progression of injury follows a pattern similar to that of a human suggesting that results reported here are relevant to clinical medicine.

Another goal of this study was to investigate a model of survival after 60% blood loss; a criterion highly relevant to battlefield medicine. However, major blood loss is not solely a battlefield occurrence. Hemorrhage is responsible for over 35% of pre-hospital deaths after traumatic injury. Of these mortalities, more than 40% occur within the first 24 hours [1]. We selected the most humane methodologies available that still allowed us to address the severity of injury seen in these human cases in a clinically relevant manner (i.e. prehospital).

## Conclusion

The present study demonstrates that AGS in both the winter and summer season are resistant to liver and kidney damage and systemic inflammation after I/R relative to the laboratory rat. This resistance to I/R injury in AGS is not dependent on their ability to cool during insult. The results indicate that an altered metabolic response associated with a lower lactate∶glucose ratio and inflammatory response following I/R may contribute to the AGS's protection. More studies are needed to examine the role of metabolism as a mechanism of I/R tolerance in the AGS.

## Supporting Information

Figure S1 Changes in circulating CPK and LDH levels before and 24 hours after cardiac arrest. Dark bars indicate baseline values. Light bars are values after CA. Raw data is shown as mean ± SEM. n = 5–6 for all groups except AGS SCA CPK n = 2 and rat CA LDH n = 3.
(TIF)

Figure S2 Multiple foci of ischemic necrosis in rat but not AGS liver after cardiac arrest. Arrows indicate areas of necrotic cells, 4× magnification.
(TIF)

Figure S3 Small intestine remains undamaged after hemorrhagic shock. Small intestine mucosal layer did not sustain damage three hours after HS as assessed by villi length (top) and histological analysis (bottom). Data shown as mean±SEM; *p<0.05, Tukey Rats versus AGS-EU and AGS-IBA. Naive values for villi length were 758.39±48.61, 720.50±28.16, 611.23±28.67 µm; damage score 0.21±0.04, 0.13±0.10, 1.26±0.27; for AGS-EU, AGS-IBA, and rats.
(TIF)

Table S1 Experimental groups for CA and HS experiments.
(DOCX)

Table S2 Characteristics of AGS subjected to CA.
(DOCX)

Table S3 Characteristics of AGS subjected to SCA.
(DOCX)

Table S4 Characteristics of AGS undergoing HS during the winter (IBA) season.
(DOCX)

Table S5 Characteristics of AGS undergoing HS during the summer (euthermic) season.
(DOCX)

Table S6 Characteristics of AGS undergoing SHS during the winter (IBA) season.
(DOCX)

Table S7 Characteristics of AGS undergoing SHS during the summer (euthermic) season.
(DOCX)

Table S8 Numerical scoring system used for quantitative histological analysis of tissues.
(DOCX)

Table S9 Organ-specific histopathologic changes examined for quantitative histological analysis of tissues.
(DOCX)

Table S10 Histopathology organ damage scores for CA experiments.
(DOCX)

Table S11 Histopathology organ damage scores for HS experiments.
(DOCX)

Table S12 Plasma cytokine concentration values (pg/mL) prior to hemorrhage and three hours after hemorrhage used to determine fold change after global I/R.
(DOCX)
