# Supplementary figures and images for: Resistance to Systemic Inflammation and Multi Organ Damage after Global Ischemia/Reperfusion in the Arctic Ground Squirrel
Source: PLoS One. 2014 Apr 11;9(4):e94225. doi: 10.1371/journal.pone.0094225 (PMC3984146; doi:10.1371/journal.pone.0094225)

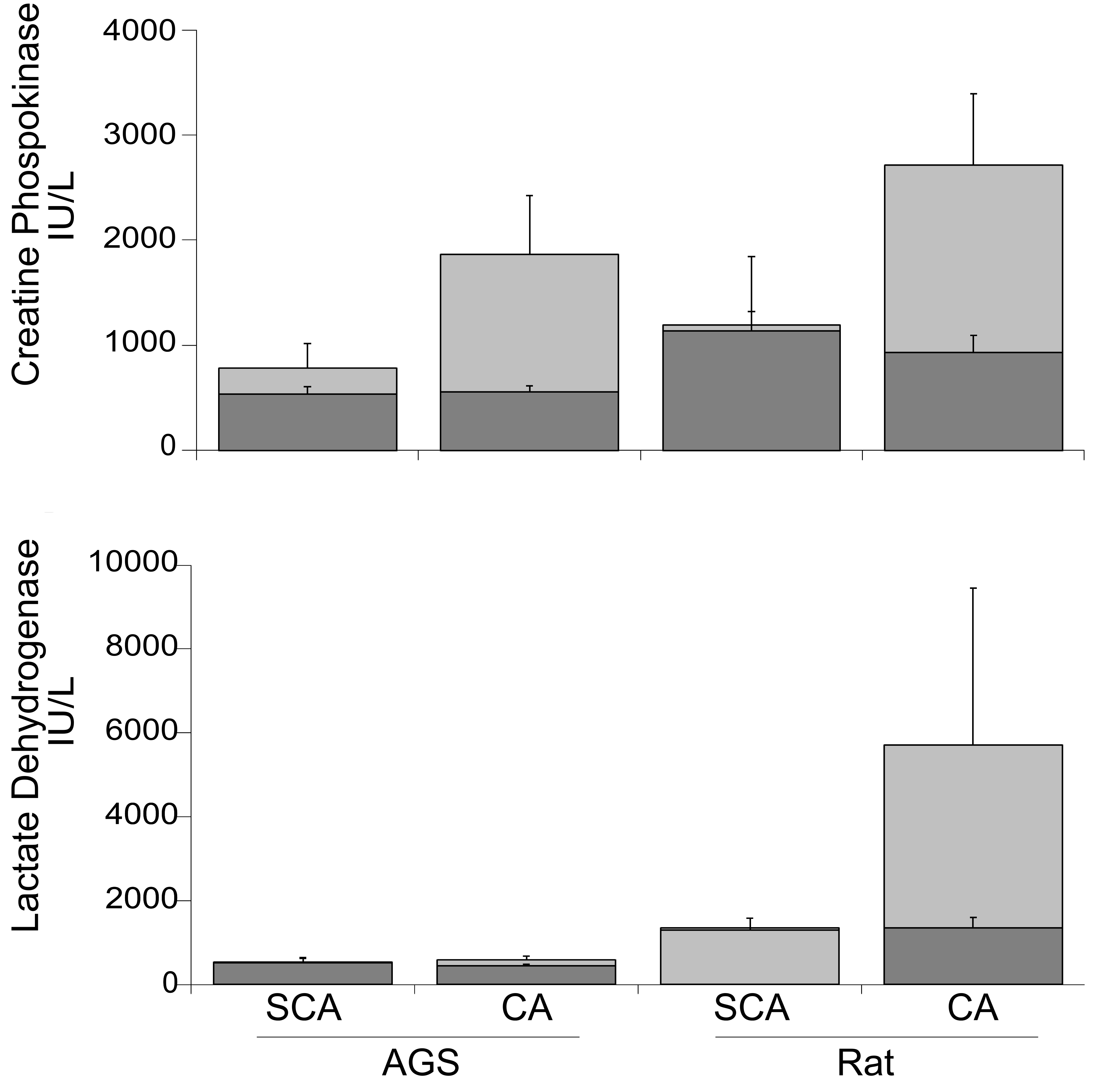

Supplement: Figure S1 — Changes in circulating CPK and LDH levels before and 24 hours after cardiac arrest. Dark bars indicate baseline values. Light bars are values after CA. Raw data is shown as mean ± SEM. n = 5–6 for all groups except AGS SCA CPK n = 2 and rat CA LDH n = 3. (TIF) [file pone.0094225.s001.tif]

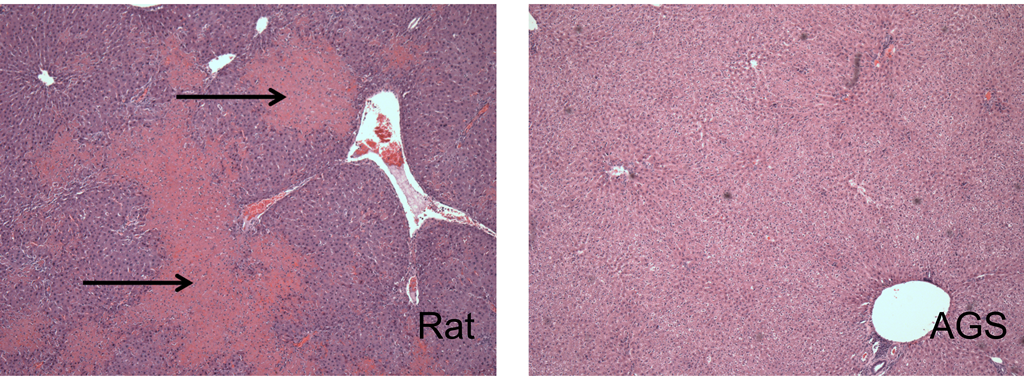

Supplement: Figure S2 — Multiple foci of ischemic necrosis in rat but not AGS liver after cardiac arrest. Arrows indicate areas of necrotic cells, 4× magnification. (TIF) [file pone.0094225.s002.tif]

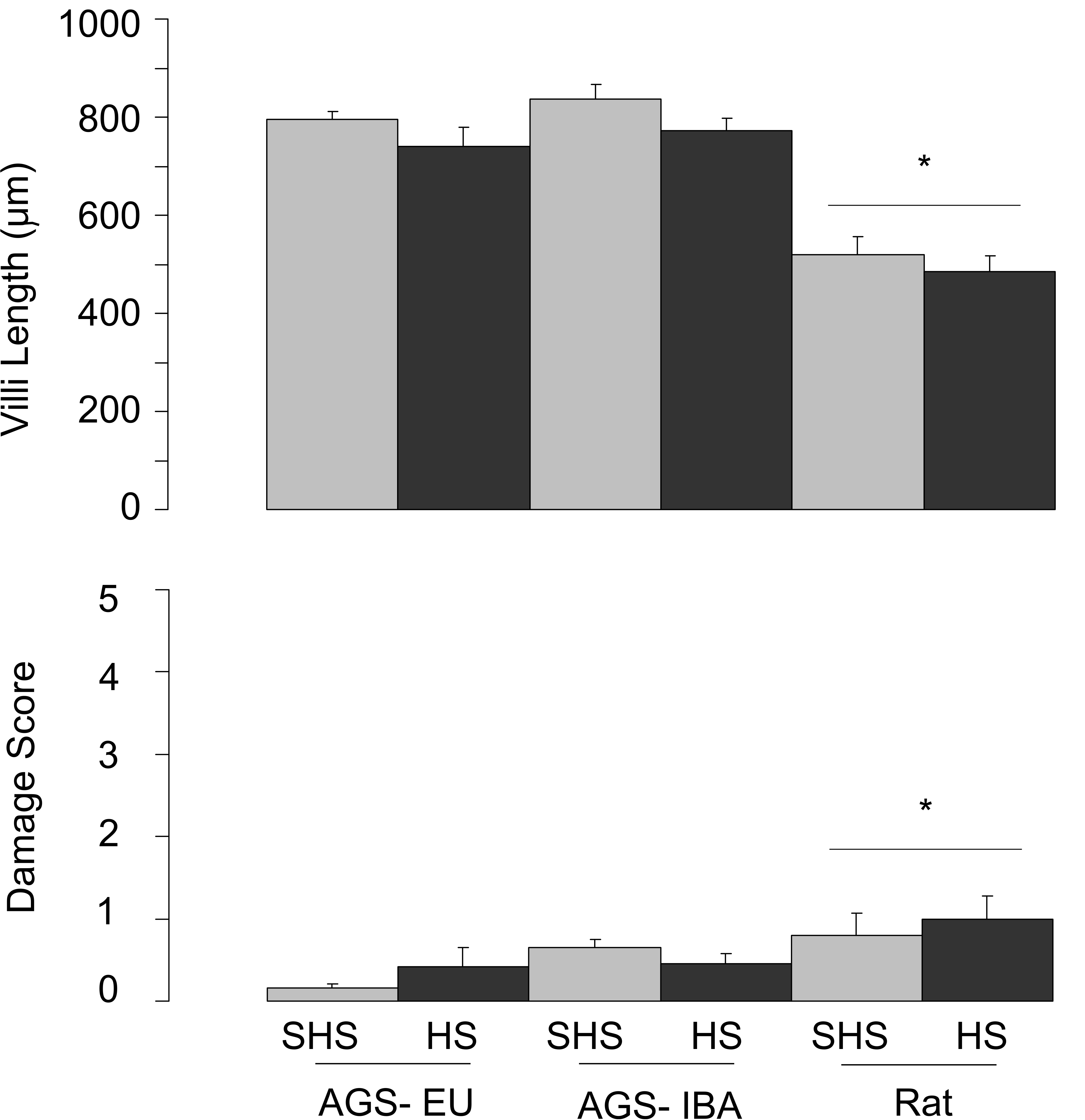

Supplement: Figure S3 — Small intestine remains undamaged after hemorrhagic shock. Small intestine mucosal layer did not sustain damage three hours after HS as assessed by villi length (top) and histological analysis (bottom). Data shown as mean±SEM; *p<0.05, Tukey Rats versus AGS-EU and AGS-IBA. Naive values for villi length were 758.39±48.61, 720.50±28.16, 611.23±28.67 µm; damage score 0.21±0.04, 0.13±0.10, 1.26±0.27; for AGS-EU, AGS-IBA, and rats. (TIF) [file pone.0094225.s003.tif]
