# Supplementary material for: Resistance to Systemic Inflammation and Multi Organ Damage after Global Ischemia/Reperfusion in the Arctic Ground Squirrel
Source: PLoS One. 2014 Apr 11;9(4):e94225. doi: 10.1371/journal.pone.0094225 (PMC3984146; doi:10.1371/journal.pone.0094225)
Supplement: Table S1 — Experimental groups for CA and HS experiments. (DOCX) [file pone.0094225.s004.docx]

Supporting Table 1.Experimental groups for CA and HS experiments

| **Experiment** | **Group** | **Treatment** | **n**  **available** | **n**  **per parameter** | **n**  **died*** |
| --- | --- | --- | --- | --- | --- |
| CA | Rat | naive | 8 | 8 | 0 |
|  |  | CA | 6 | 4-5 | 0 |
|  |  | SCA | 6 | 4-5 | 0 |
|  | AGS | naive | 8 | 8 | 0 |
|  |  | CA | 6 | 4-5 | 0 |
|  |  | SCA | 6 | 4-5 | 0 |
| HS | Rat | naive | 8 | 8 | 0 |
|  |  | HS | 8 | 4-8 | 0 |
|  |  | SHS | 8 | 4-8 | 0 |
|  | AGS-EU | naive | 7 | 7 | 0 |
|  |  | HS | 6 | 4-6 | 0 |
|  |  | SHS | 7 | 4-7 | 0 |
|  | AGS-IBA | naive | 8 | 8 | 0 |
|  |  | HS | 7 | 4-7 | 0 |
|  |  | SHS | 8 | 4-8 | 0 |
| HS-isovolumetric | AGS-EU | HS | 4 | 4 | 3 |
|  | AGS-IBA | HS | 4 | 4 | 4 |
| HS-72 hour survival | Rat | HS | 4 | 4 | 4 |
|  | AGS-EU | HS | 4 | 4 | 0 |
|  | AGS-IBA | HS | 4 | 4 | 0 |

*Animals that died due to experimental treatment and were not euthanized under anesthesia at the end of the protocol. Of all of the animals that died outside of euthanasia, only two rats (72-hour survival) died while not under anesthesia. CA: cardiac arrest, SCA: sham cardiac arrest, HS: Hemorrhagic shock, SHS: sham hemorrhagic shock, AGS: arctic ground squirrel, EU: euthermic (summer), IBA: interboutarousal (winter).
