# Supplementary material for: Resistance to Systemic Inflammation and Multi Organ Damage after Global Ischemia/Reperfusion in the Arctic Ground Squirrel
Source: PLoS One. 2014 Apr 11;9(4):e94225. doi: 10.1371/journal.pone.0094225 (PMC3984146; doi:10.1371/journal.pone.0094225)
Supplement: Table S2 — Characteristics of AGS subjected to CA. (DOCX) [file pone.0094225.s005.docx]

**Supporting Table 2. Characteristics of AGS subjected to CA.**

| Animal number | 09-03 | 09-04 | 09-47 | 09-35 | 09-84 | 09-10 | 09-67 |
| --- | --- | --- | --- | --- | --- | --- | --- |
| Experimental Group | CA | | | | | | |
| Age | Adult | Adult | Adult | Adult | Adult | Adult | Adult |
| Sex | Male | Male | Female | Female | Male | Male | Male |
| Mass (g) | 724 | 601 | 491 | 569 | 563 | 564 | 628 |
| Experiment day | 26-May-10 | 27-May-10 | 6-Jun-10 | 7-Jun-10 | 11-Jun-10 | 14-Jun-10 | 28-Jun-10 |
| Last day of torpor during previous season | 7-Apr-10 | no torpor | 26-Mar-10 | 13-Mar-10 | 14-Mar-10 | 19-Feb-10 | no torpor |

T_b_ of all animals was 37±0.5°C at the start of HS experiment.
