# Supplementary material for: Resistance to Systemic Inflammation and Multi Organ Damage after Global Ischemia/Reperfusion in the Arctic Ground Squirrel
Source: PLoS One. 2014 Apr 11;9(4):e94225. doi: 10.1371/journal.pone.0094225 (PMC3984146; doi:10.1371/journal.pone.0094225)
Supplement: Table S3 — Characteristics of AGS subjected to SCA. (DOCX) [file pone.0094225.s006.docx]

**Supporting Table 3. Characteristics of AGS subjected to SCA**

| Animal number | 08-64 | 08-66 | 09-18 | 09-44 | 09-05 | 09-23 |
| --- | --- | --- | --- | --- | --- | --- |
| Experimental Group | SCA | | | | | |
| Age | Adult | Adult | Adult | Adult | Adult | Adult |
| Sex | Male | Male | Male | Male | Male | Female |
| Mass (g) | 1046 | 812 | 760 | 637 | 645 | 610 |
| Experiment day | 29-Apr-10 | 18-May-10 | 4-Jun-10 | 8-Jun-10 | 10-Jun-10 | 22-Jun-10 |
| Last day of torpor during previous season | 7-Mar-10 | 24-Aug-09 | 3-Mar-10 | 14-Mar-10 | 22-Mar-10 | 12-Apr-10 |

T_b_ of all animals was 37±0.5°C at the start of HS experiment.
