# Supplementary material for: Resistance to Systemic Inflammation and Multi Organ Damage after Global Ischemia/Reperfusion in the Arctic Ground Squirrel
Source: PLoS One. 2014 Apr 11;9(4):e94225. doi: 10.1371/journal.pone.0094225 (PMC3984146; doi:10.1371/journal.pone.0094225)
Supplement: Table S4 — Characteristics of AGS undergoing HS during the winter (IBA) season. (DOCX) [file pone.0094225.s007.docx]

**Supporting Table 4. Characteristics of AGS undergoing HS during the winter (IBA) season.**

T_b_ of all animals was 37±0.5°C at the start of HS experiment.

| Animal number | 09-56 | 08-83 | 08-40 | 09-07 | 08-68 | 10-02 | 10-08 | 10-05 | 10-48 |
| --- | --- | --- | --- | --- | --- | --- | --- | --- | --- |
| Season | Winter | | | | | | | | |
| Age | Adult | Adult | Adult | Adult | Adult | Adult | Adult | Adult | Adult |
| Sex | Female | Female | Female | Male | Male | Female | Female | Female | Female |
| Mass (g) | 862 | 1123 | 1044 | 556 | 1056 | 461 | 441 | 559 | 721 |
| First day of spontaneous torpor | 18-Aug-10 | 18-Aug-10 | 17-Aug-10 | 19-Aug-10 | 4-Aug-10 | 25-Aug-10 | 22-Aug-10 | 29-Aug-10 | 2-Oct-10 |
| Experiment day | 7-Jan-11 | 11-Jan-11 | 24-Jan-11 | 25-Jan-11 | 27-Jan-11 | 1-Feb-11 | 10-Feb-11 | 17-Feb-11 | 18-Feb-11 |
| No. of spontaneous torpor bouts prior to HS | 11 | 16 | 14 | 11 | 14 | 14 | 10 | 13 | 13 |
| Average length of previous 3 torpor bouts (days) | 14.67 | 10.67 | 11.33 | 8.33 | 13.67 | 12.33 | 20.33 | 17.67 | 17.67 |
| Day in bout | 6 | 4 | 4 | 8 | 4 | 5 | 13 | 2 | 11 |
| T_b_ at induced arousal (°C) | 3 | 3.3 | 2.9 | 3.3 | 3 | 3.3 | 3.5 | 4 | 4.7 |
| Blood volume removed (% total) | 16 | 15 | 22 | 56 | 24 | 58 | 42 | 28 | 37 |
