# Supplementary material for: Resistance to Systemic Inflammation and Multi Organ Damage after Global Ischemia/Reperfusion in the Arctic Ground Squirrel
Source: PLoS One. 2014 Apr 11;9(4):e94225. doi: 10.1371/journal.pone.0094225 (PMC3984146; doi:10.1371/journal.pone.0094225)
Supplement: Table S5 — Characteristics of AGS undergoing HS during the summer (euthermic) season. (DOCX) [file pone.0094225.s008.docx]

**Supporting Table 5. Characteristics of AGS undergoing HS during the summer (euthermic) season.**

T_b_ of all animals was 37±0.5°C at the start of HS experiment.

| Animal number | 10-26 | 10-46 | 10-06 | 10-44 | 09-59 | 09-54 |
| --- | --- | --- | --- | --- | --- | --- |
| Season | Summer | | | | | |
| Age | Adult | Adult | Adult | Adult | Adult | Adult |
| Sex | Male | Male | Female | Male | Female | Female |
| Mass (g) | 856 | 549 | 580 | 619 | 591 | 673 |
| Last day of torpor during previous season | 26-Jan-11 | 10-Feb-11 | 24-Feb-11 | 23-Feb-11 | 12-Mar-11 | 5-Apr-11 |
| Experiment day | 9-Jun-11 | 14-Jun-11 | 23-Jun-11 | 24-Jun-11 | 8-Jul-11 | 14-Jul-11 |
| Blood volume removed (% total) | 22 | 28 | 62 | 40 | 34 | 38 |
