# Supplementary material for: Resistance to Systemic Inflammation and Multi Organ Damage after Global Ischemia/Reperfusion in the Arctic Ground Squirrel
Source: PLoS One. 2014 Apr 11;9(4):e94225. doi: 10.1371/journal.pone.0094225 (PMC3984146; doi:10.1371/journal.pone.0094225)
Supplement: Table S7 — Characteristics of AGS undergoing SHS during the summer (euthermic) season. (DOCX) [file pone.0094225.s010.docx]

**Supporting Table 7. Characteristics of AGS undergoing SHS during the summer (euthermic) season.**

| Animal number | 09-76 | 09-83 | 10-16 | 10-27 | 10-14 | 09-68 | 10-30 |
| --- | --- | --- | --- | --- | --- | --- | --- |
| Season | Summer | | | | | | |
| Age | Adult | Adult | Adult | Adult | Adult | Adult | Adult |
| Sex | Female | Male | Male | Male | Female | Female | Female |
| Mass (g) | 1036 | 554 | 464 | 658 | 617 | 833 | 446 |
| Last day of torpor during previous season | 10-Nov-10 | 10-Feb-11 | 20-Feb-11 | 09-Feb-11 | 28-Feb-11 | 5-Jan-11 | 27-Feb-11 |
| Experiment day | 7-Jun-11 | 10-Jun-11 | 16-Jun-11 | 21-Jun-11 | 28-Jun-11 | 29-Jun-11 | 15-Jul-11 |

T_b_ of all animals was 37±0.5°C at the start of HS experiment.
