# Supplementary material for: Resistance to Systemic Inflammation and Multi Organ Damage after Global Ischemia/Reperfusion in the Arctic Ground Squirrel
Source: PLoS One. 2014 Apr 11;9(4):e94225. doi: 10.1371/journal.pone.0094225 (PMC3984146; doi:10.1371/journal.pone.0094225)
Supplement: Table S8 — Numerical scoring system used for quantitative histological analysis of tissues. (DOCX) [file pone.0094225.s011.docx]

Supporting Table 8. Numerical scoring system used for quantitative histological analysis of tissues.

| **Grade** | **Score** |
| --- | --- |
| Normal | 0 |
| Minimal Focal | 0.25 |
| Minimal Multifocal | 0.5 |
| Minimal Locally Extensive | 0.75 |
| Minimal | 1 |
| Mild Focal | 1.25 |
| Mild Multifocal | 1.5 |
| Mild Locally Extensive | 1.75 |
| Mild | 2 |
| Moderate Focal | 2.25 |
| Moderate Multifocal | 2.5 |
| Moderate Locally Extensive | 2.75 |
| Moderate | 3 |
| Marked Focal | 3.25 |
| Marked Multifocal | 3.5 |
| Marked Locally Extensive | 3.75 |
| Marked | 4 |
