# Supplementary material for: Resistance to Systemic Inflammation and Multi Organ Damage after Global Ischemia/Reperfusion in the Arctic Ground Squirrel
Source: PLoS One. 2014 Apr 11;9(4):e94225. doi: 10.1371/journal.pone.0094225 (PMC3984146; doi:10.1371/journal.pone.0094225)
Supplement: Table S9 — Organ-specific histopathologic changes examined for quantitative histological analysis of tissues. (DOCX) [file pone.0094225.s012.docx]

Supporting Table 9. Organ-specific histopathologic changes examined for quantitative histological analysis of tissues.

| **Primary Tissue** | **Secondary Level** | **Change** |
| --- | --- | --- |
|  |  |  |
| **Skeletal Muscle** | **N/A** | Fiber Atrophy |
|  |  | Granular Degeneration |
|  |  | Lymphoid Cuffing |
|  |  | Mixed Cell Reaction |
|  |  | Parasitic Cyst |
| **Heart** | **Atria** | Hyaline Fiber Degeneration |
|  |  | Interstitial Edema |
|  |  | Lymphoid Reaction |
|  |  | Mixed Cell Reaction |
|  |  | Pyknosis |
|  |  | Vesiculation Fiber Nuclei |
|  | **Left Ventricle** | Granular Degeneration |
|  |  | Hyaline Fiber Degeneration |
|  |  | Interstitial Edema |
|  |  | Interstitial Fibrosis |
|  |  | Lymphoid Reaction |
|  |  | Mixed Cell Reaction |
|  | **Right Ventricle** | Hyaline Fiber Degeneration |
|  |  | Interstitial Edema |
|  |  | Lymphoid Reaction |
|  | **Vessels** | Interstitial Edema |
| **Lung** | **Bronchi** | Peribronchial Lymphoid Reaction |
|  | **Parenchyma** | Arteriolar Hypertrophy |
|  |  | Foreign Body |
|  |  | Congestion |
|  |  | Granuloma |
|  |  | Increased Vascular Leukocytes |
|  |  | Mixed Cell Reaction |
|  |  | Perivascular Lymphoid Reaction |
| **Spleen** | **Architecture** | Follicular Hyperplasia |
|  |  | Marginal Zone Hypertrophy |
|  |  | Arteriole Cuff Atrophy |
|  |  | Follicular Hypertrophy |
|  |  | Mantle Cell Cuff Hypertrophy |
|  | **Lymph Compartments** | Follicular Involution |
|  |  | Increased Volume of PALS |
|  |  | Reduced Cell Density of PALS |
|  |  | Reduced Cell Density of Follicles |
|  |  | Reduced Volume of PALS |
|  | **Sinus Compartment** | Erythrophagocytosis |
|  |  | Erythropoiesis |
|  |  | Sinus Atrophy |
|  |  | Lymphocytosis |
|  |  | Lymphoid Cuffing of Trabecula |
| **Liver** | **Architecture** | Accentuation of Zonation |
|  |  | Lobar Infarction |
|  |  | Lobular Necrosis |
|  |  | Peribiliary Fibrosis |
|  |  | Prominent Biliary Tracts |
|  | **Biliary Ducts / Portal Areas** | Periportal Lymph Cuffs |
|  |  | Periportal Mixed Cell Cuffs |
|  | **Hepatocellular Cytoplasm** | Decreased Perivenous Volume |
|  |  | Decreased Perivenous Density |
|  |  | Increased Perivenous Density |
|  |  | Increased Portal Density |
|  |  | Increased Perivenous Homogeneity |
|  |  | Increased Portal Homogeneity |
|  |  | Decreased Portal Volume |
|  |  | Increased Portal Volume |
|  |  | Decreased Midzone Volume |
|  |  | Midzone Vacuolation |
|  | **Hepatocellular Nuclei** | Anisokaryosis |
|  |  | Binucleation |
|  |  | Multinucleation |
|  |  | Pyknosis |
|  |  | Vesiculation |
|  |  | Zonal Necrosis |
|  | **Interstitium** | Extramedullary Hematopoiesis |
|  |  | Granulocytic Reaction |
|  |  | Granulomatous Reaction |
|  |  | Increased Endothelial Nuclei |
|  |  | Increased Endothelial Prom |
|  |  | Kupffer Cell Pigmentation |
|  |  | Lymphoid Reaction |
| **Kidney** | **Architecture** | Cortical Scarring |
|  |  | Tubular Dilation |
|  |  | Cortical Necrosis |
|  | **Glomeruli** | Senescence |
|  | **Outer Stripe Cortex** | Anisokaryosis |
|  |  | Primary Injury |
|  |  | Secondary Injury |
|  |  | Cortical Scarring |
|  |  | Protein Casts |
|  |  | Necrosis |
|  | **Inner Stripe Cortex** | Primary Injury |
|  |  | Protein Casts |
|  |  | Secondary Injury |
|  |  | Intranephronic Calculosis |
|  |  | Anisokaryosis |
|  |  | Necrosis |
|  | **Medulla** | Primary Injury |
|  |  | Protein Casts |
|  |  | Intranephronic Calculosis |
|  | **Interstitium** | Arter Embolism |
|  |  | Lymphoid Cuffing |
|  |  | Reticulin Sclerosis |
| **Small Intestine** | **N/A** | Submucosal Lymph Follicles |
|  |  | Epitheliotropic Lymphoid Reaction |
|  |  | Increased Epithelial Lymphs |
|  |  | Increased Subepithelial Lymphs |
| **Large Intestine** | **N/A** | Increased Subepithelial Lymphs |
|  |  | Submucosal Cell Reaction |
|  |  | Submucosal Lymph Follicles |
