# Supplementary material for: Resistance to Systemic Inflammation and Multi Organ Damage after Global Ischemia/Reperfusion in the Arctic Ground Squirrel
Source: PLoS One. 2014 Apr 11;9(4):e94225. doi: 10.1371/journal.pone.0094225 (PMC3984146; doi:10.1371/journal.pone.0094225)
Supplement: Table S10 — Histopathology organ damage scores for CA experiments. (DOCX) [file pone.0094225.s013.docx]

**Supporting Table 10. Histopathology organ damage scores for CA experiments.**

|  | AGS | | | Rat | | |
| --- | --- | --- | --- | --- | --- | --- |
|  | Naive | SCA | CA | Naive | SCA | CA |
| Small Intestine | 1.36±0.39 | 0.54±0.21 | 0.71±0.19 | n/a | 0.63±0.24 | 0.45±0.12 |
| Large Intestine | 0.86±0.36 | 0.67±0.42 | 0.25±0.25 | n/a | 0.06±0.06 | 0.45±1.01 |
| Lung | 2.72±0.41 | 2.21±0.74 | 3.63±0.36 | 1.15±0.31 | 1.38±0.21 | 1.15±0.36 |
| Kidney | 0.42±0.22 | 3.33±1.62 | 0.25±0.17 | 7.81±1.19 | 1.00±0.42 | 2.15±0.39 |
| Spleen | 2.61±0.43 | 3.92±0.93 | 3.50±0.93 | 12.19±1.70 | 6.87±1.07 | 8.00±0.78 |
| Liver | 4.75±0.98 | 7.83±1.23 | 7.54±1.35 | 2.15±0.87 | 3.68±0.48 | 6.70±3.72 |
| Heart | 0.64±0.43 | 0.46±0.26 | 1.83±0.85 | n/a | 1.13±0.33 | 0.25±0.25 |

Significant histopathology due to CA treatment versus sham (SCA) was not found in any organ examined for either species. Data are shown as mean±SEM, n=4-9 for all groups.
