# Supplementary material for: Resistance to Systemic Inflammation and Multi Organ Damage after Global Ischemia/Reperfusion in the Arctic Ground Squirrel
Source: PLoS One. 2014 Apr 11;9(4):e94225. doi: 10.1371/journal.pone.0094225 (PMC3984146; doi:10.1371/journal.pone.0094225)
Supplement: Table S11 — Histopathology organ damage scores for HS experiments. (DOCX) [file pone.0094225.s014.docx]

**Supporting Table 11. Histopathology organ damage scores for HS experiments.**

|  | AGS-EU | | | AGS-IBA | | | Rat | | |
| --- | --- | --- | --- | --- | --- | --- | --- | --- | --- |
|  | Naïve | SHS | HS | Naive | SHS | HS | Naive | SHS | HS |
| Intestine | 0.39±0.26 | 0.93±0.42 | 0.08±0.08 | 0.04±0.03 | 0.19±0.19 | 0.03±0.03 | 0.84±0.22 | 2.31±0.53 | 1.94±0.39 |
| Lung | 1.18±0.44 | 3.04±1.29 | 2.88±0.90 | 0.94±0.25 | 1.22±0.45 | 2.19±0.60 | 1.09±0.42 | 0.94±0.34 | 1.66±0.54 |
| Kidney | 4.75±2.02 | 4.07±1.00 | 3.79±0.84 | 1.16±0.63 | 2.13±0.68 | 3.19±1.19 | 8.25±1.12 | 9.31±20.5 | 11.97±2.74 |
| Spleen | 3.11±1.15 | 2.86±0.47 | 3.75±0.85 | 3.59±0.45 | 3.25±0.70 | 3.12±1.10 | 11.44±1.59 | 11.75±1.10 | 11.81±1.31 |
| Liver | 3.54±0.96 | 5.21±1.66 | 6.54±1.61 | 4.50±0.70 | 5.69±1.88 | 5.03±0.56 | 3.03±1.21 | 5.06±0.79 | 5.94±1.09 |

Significant histopathology due to HS treatment was not found in any organ examined for either species. Data are shown as mean ±SEM. n=6-8 for all groups. HS: Hemorrhagic shock, SHS: sham hemorrhagic shock, AGS: arctic ground squirrel, EU: euthermic (summer), IBA: interboutarousal (winter).
