# Supplementary material for: Resistance to Systemic Inflammation and Multi Organ Damage after Global Ischemia/Reperfusion in the Arctic Ground Squirrel
Source: PLoS One. 2014 Apr 11;9(4):e94225. doi: 10.1371/journal.pone.0094225 (PMC3984146; doi:10.1371/journal.pone.0094225)
Supplement: Table S12 — Plasma cytokine concentration values (pg/mL) prior to hemorrhage and three hours after hemorrhage used to determine fold change after global I/R. (DOCX) [file pone.0094225.s015.docx]

**Supporting Table 12. Plasma cytokine concentration values (pg/mL) prior to hemorrhage and three hours after hemorrhage used to determine fold change after global I/R.**

|  | AGS-EU | | | | AGS-IBA | | | | Rat | | | |
| --- | --- | --- | --- | --- | --- | --- | --- | --- | --- | --- | --- | --- |
|  | SHS | | HS | | SHS | | HS | | SHS | | HS | |
|  | Prior | After | Prior | After | Prior | After | Prior | After | Prior | After | Prior | After |
| IL-1 alpha | 72.81±104.73 | 58.93±30.01 | 102.51±68.94 | 74.29±63.30 | 56.48±20.14 | 60.97±28.35 | 53.46±33.17 | 43.45±28.98 | 21.82±2.45 | 23.10±3.27 | 20.17±1.20 | 92.91±22.74 |
| IL-1 beta | 15.26±20.08 | 20.01±17.04 | 5.25±1.79 | 6.44±2.05 | 15.20±4.10 | 16.35±7.28 | 15.10±3.55 | 10.51±3.25 | 30.54±3.81 | 63.20±16.33 | 29.78±2.59 | 113.76±22.68 |
| IL-6 | 16.83±8.09 | 20.53±5.58 | 13.85±2.73 | 10.95±2.07 | 16.65±3.10 | 16.15±2.90 | 18.81±5.92 | 17.91±9.10 | 35.07±2.14 | 139.09±29.95 | 31.11±2.95 | 1170.82±643.03 |
| IL-10 | 68.02±17.31 | 54.81±4.34 | 61.93±3.95 | 49.86±3.83 | 60.60±7.60 | 54.56±11.88 | 67.20±5.33 | 46.43±4.41 | 108.87±7.69 | 121.00±14.74 | 96.66±5.80 | 404.17±167.96 |
| TNF-alpha | 2.72±2.64 | 3.15±0.80 | 1.85±0.56 | 2.5±0.74 | 4.26±1.20 | 3.33±1.51 | 3.04±0.59 | 3.27±0.60 | 5.82±0.67 | 5.58±1.04 | 6.10±0.71 | 46.52±13.55 |
| INF- gamma | 3.07±2.03 | 3.98±1.29 | 7.67±4.50 | 3.05±1.65 | 9.04±5.44 | 3.77±1.82 | 12.91±9.60 | 6.52±3.98 | 4.29±0.38 | 3.53±0.60 | 3.55±0.36 | 12.78±3.58 |

Difference in cytokine levels was determined from fold change shown in Figure 8. Due to the chance of species differences in antibody binding affinity, statistical analysis was not preformed on the raw numbers. Data are shown as mean ±SEM. n=6-8 for all groups. HS: Hemorrhagic shock, SHS: sham hemorrhagic shock, AGS: arctic ground squirrel, EU: euthermic (summer), IBA: interboutarousal (winter).
